# Comparison of Methods to Estimate Low-Density Lipoprotein Cholesterol in Patients With High Triglyceride Levels

**DOI:** 10.1001/jamanetworkopen.2021.28817

**Published:** 2021-10-28

**Authors:** Aparna Sajja, Jihwan Park, Vasanth Sathiyakumar, Bibin Varghese, Vincent A. Pallazola, Francoise A. Marvel, Krishnaji Kulkarni, Alagarraju Muthukumar, Parag H. Joshi, Eugenia Gianos, Benjamin Hirsh, Guy Mintz, Anne Goldberg, Pamela B. Morris, Garima Sharma, Roger S. Blumenthal, Erin D. Michos, Wendy S. Post, Mohamed B. Elshazly, Steven R. Jones, Seth S. Martin

**Affiliations:** 1 Ciccarone Center for the Prevention of Cardiovascular Disease, Division of Cardiology, Department of Medicine, Johns Hopkins University School of Medicine, Baltimore, Maryland; 2 Department of Epidemiology, Johns Hopkins Bloomberg School of Public Health, Baltimore, Maryland; 3 VAP Diagnostics Lab, Birmingham, Alabama; 4 Department of Pathology, University of Texas Southwestern Medical Center, Dallas; 5 Division of Cardiology, Department of Internal Medicine, University of Texas /Southwestern Medical Center, Dallas; 6 Department of Cardiology, North Shore University Hospital, Northwell Health, Zucker School of Medicine, New York, New York; 7 Division of Endocrinology, Metabolism, and Lipid Research, Washington University School of Medicine in St Louis, St Louis, Missouri; 8 Department of Cardiology, Medical University of South Carolina, Columbia; 9 Welch Center for Prevention, Epidemiology, and Clinical Research, Department of Epidemiology, Johns Hopkins Bloomberg School of Public Health, Baltimore, Maryland; 10 Department of Cardiovascular Medicine, Weill Cornell Medicine-Qatar, Education City, Doha, Qatar

## Abstract

**Importance:**

Low-density lipoprotein cholesterol (LDL-C) is typically estimated with the Friedewald or Martin/Hopkins equation; however, if triglyceride levels are 400 mg/dL or greater, laboratories reflexively perform direct LDL-C (dLDL-C) measurement. The use of direct chemical LDL-C assays and estimation of LDL-C via the National Institutes of Health Sampson equation are not well validated, and data on the accuracy of LDL-C estimation at higher triglyceride levels are limited.

**Objective:**

To compare an extended Martin/Hopkins equation for triglyceride values of 400 to 799 mg/dL with the Friedewald and Sampson equations.

**Design, Setting, and Participants:**

This cross-sectional study evaluated consecutive patients at clinical sites across the US with patient lipid distributions representative of the US population in the Very Large Database of Lipids from January 1, 2006, to December 31, 2015, with triglyceride levels of 400 to 799 mg/dL. Data analysis was performed from November 9, 2020, to March 23, 2021.

**Main Outcomes and Measures:**

Accuracy in LDL-C classification according to guideline-based categories and absolute errors between estimated LDL-C and dLDL-C levels. Patients were randomly assigned 2:1 to derivation and validation data sets. Levels of dLDL-C were measured by vertical spin-density gradient ultracentrifugation. The LDL-C levels were estimated using the Friedewald method, with a fixed ratio of triglycerides to very low-density lipoprotein cholesterol (VLDL-C ratio of 5:1), extended Martin/Hopkins equation with a flexible ratio, and Sampson equation with VLDL-C estimation by multiple least-squares regression.

**Results:**

A total of 111 939 patients (mean [SD] age, 52 [13] years; 65.0% male) with triglyceride levels of 400 to 799 mg/dL were included, representing 2.2% of 5 081 680 patients in the database. Across all individual guideline LDL-C classes (<40, 40-69, 70-99, 100-129, 130-159, 160-189, and ≥190), estimation of LDL-C by the extended Martin/Hopkins equation was most accurate (62.1%) compared with the Friedewald (19.3%) and Sampson (40.4%) equations. In classifying LDL-C levels less than 70 mg/dL across all triglyceride strata, the extended Martin/Hopkins equation was most accurate (67.3%) compared with Friedewald (5.1%) and Sampson (26.4%) equations. In addition, for classifying LDL-C levels less than 40 mg/dL across all triglyceride strata, the extended Martin/Hopkins equation was most accurate (57.2%) compared with the Friedewald (4.3%) and Sampson (14.4%) equations. However, considerable underclassification of LDL-C occurred. The magnitude of error between the Martin/Hopkins equation estimation and dLDL-C was also smaller: at LDL-C levels less than 40 mg/dL, 2.7% of patients had 30 mg/dL or greater differences between dLDL-C and estimated LDL-C using the Martin/Hopkins equation compared with the Friedewald (92.5%) and Sampson (38.7%) equations.

**Conclusions and Relevance:**

In this cross-sectional study, the extended Martin/Hopkins equation offered greater LDL-C accuracy compared with the Friedewald and Sampson equations in patients with triglyceride levels of 400 to 799 mg/dL. However, regardless of method used, caution is advised with LDL-C estimation in this triglyceride range.

## Key Points

**Question:**

What is the accuracy of low-density lipoprotein cholesterol (LDL-C) estimation when comparing the Friedewald, extended Martin/Hopkins, and Sampson equations with LDL-C measured through ultracentrifugation at triglyceride levels of 400 to 799 mg/dL?

**Findings:**

In this cross-sectional study of 111 939 patients, across all individual guideline LDL-C classes from <40 to ≥190 mg/dL, estimation of LDL-C with the extended Martin/Hopkins equation was most accurate (62.1%) compared with the Friedewald (19.3%) and Sampson (40.4%) equations.

**Meaning:**

These results suggest that the extended Martin/Hopkins method offers greater accuracy compared with the Friedewald and Sampson equations at triglyceride levels of 400 to 799 mg/dL, with less underestimation at low LDL-C levels compared with the Friedewald and Sampson equations.

## Teaser

This cross-sectional study of patients with lipid distributions representative of the US population compares the accuracy of 3 low-density lipoprotein level estimation methods with triglyceride levels of 400 to 799 mg/dL.

## Introduction

Low-density lipoprotein cholesterol (LDL-C) has long been clinically important in cardiovascular risk assessment and treatment decision-making, with the 2018 American Heart Association/American College of Cardiology (AHA/ACC) Cholesterol Guideline focusing on LDL-C as a primary target.^1^ Furthermore, with increasing obesity and diabetes rates and new LDL-C–lowering therapies, the clinical picture of very high levels of triglycerides and reduced LDL-C levels has become more common.^2,3,4^

Current methods of LDL-C estimation, including the Friedewald equation, which assumes a fixed ratio of triglycerides to very low-density lipoprotein cholesterol (VLDL-C), and the Martin/Hopkins equation, with an adjustable ratio of triglycerides to VLDL-C based on triglyceride and non–high-density lipoprotein cholesterol (HDL-C) levels, are validated for triglyceride levels less than 400 mg/dL. Data on the accuracy of LDL-C estimation at higher triglyceride levels in large, modern cohorts are limited.^5,6^

If triglyceride levels are 400 mg/dL or higher, laboratories commonly default to error messages that LDL-C cannot be calculated, and direct chemical LDL-C assays are reflexively performed. However, direct chemical LDL-C assays are not well validated in this setting. These direct tests do not use ultracentrifugation to isolate LDL-C but instead use various proprietary chemicals that lack standardization and add time and cost.^7^

Recently, the National Institutes of Health, or Sampson, LDL-C equation was developed using multiple least-squares regression for VLDL-C estimation with a triglyceride term divided by a fixed factor, which was proposed to extend LDL-C calculation to triglyceride levels of 800 mg/dL. An extended Martin/Hopkins equation with its adaptable ratio of triglycerides VLDL-C may be more accurate at high triglyceride levels than the Friedewald or Sampson equations, which have fixed terms. The objective of our study was to compare the accuracy of LDL-C estimation using these 3 methods with LDL-C levels measured through ultracentrifugation at triglyceride levels of 400 to 799 mg/dL.

## Methods

### Study Population

In this cross-sectional study, we examined data from the Very Large Database of Lipids, which has been described in detail previously.^8^ The lipid panels in this database are from thousands of practitioners across a wide variety of clinical sites in the US. Most patients who contributed lipid panels to the database were seen at primary care clinics, whereas the remaining patients were seen at specialized clinics, such as lipid clinics, or inpatient units at university-based and community hospitals. Lipid distributions of patients in the Very Large Database of Lipids are nearly identical to those of the National Health and Nutrition Examination Survey, a nationally representative population-based cohort. The Johns Hopkins Institutional Review Board declared our study exempt because we used deidentified data routinely collected during lipid determinations. Therefore, no informed consent was required. This study followed the Strengthening the Reporting of Observational Studies in Epidemiology (STROBE) reporting guideline.

We analyzed all patients from the second harvest of the Very Large Database of Lipids with triglyceride levels of 400 to 799 mg/dL,^8^ with the upper limit of triglyceride levels chosen to respect the intended upper limit of the Sampson equation. To reflect real-world practice, we did not exclude patients based on fasting status for our main analysis. Samples were obtained by the Vertical Auto Profile (VAP) Diagnostics Laboratory from January 1, 2006, to December 31, 2015. Deidentified data were transferred to the academic investigators. Data analysis was performed from November 9, 2020, to March 23, 2021.

### Lipid Measurements

VAP, a rapid ultracentrifugation technique with a single vertical spin density gradient, was used to directly measure total cholesterol, LDL-C, VLDL-C, HDL-C, and other lipoprotein parameters.^5,9^ Triglyceride levels were directly measured with the Architect C-8000 system (Abbott Laboratories). Accuracy of VAP was reviewed by yearly random split-sample comparisons with β quantification at the Washington University Core Laboratory for Clinical Studies; directly measured triglyceride concentrations were compared with samples from University of Alabama School of Medicine for quality assessment.

### Lipid Estimations

For the Martin/Hopkins equation, we randomly assigned two-thirds of patients to a derivation data set and one-third to a validation data set. Per the Martin/Hopkins equation, LDL-C was calculated as total cholesterol − HDL-C − triglycerides/prior adjustable factor.^5^ An extended version of this Martin/Hopkins equation (LDL-C_E_) at triglyceride levels of 400 to 799 mg/dL was calculated using a strata-specific median ratio of triglycerides to VLDL-C based on 40 triglyceride and 6 non–HDL-C categories (240-cell stratification). Strata-specific median ratios of triglycerides to VLDL-C from the derivation data set were applied to the validation data set to generate LDL-C estimates.

We stratified data based on triglyceride and non–HDL-C levels because of performance in explaining variance in the ratio of triglycerides to VLDL-C compared with other parameters and the ability to capture information on the elements from the standard lipid panel.^5^ We generated 2-dimensional tables of median ratios of triglycerides to VLDL-C by varying the number of triglyceride and non–HDL-C strata based on accepted cut points (non–HDL-C: <100, 100-129, 130-159, 160-189, 190-219, and ≥220 mg/dL) and using 240, 560, and 1040 cells. Cell counts and IQRs are given in eTable 1 in the Supplement. We focused on 240-cell results in our study because the overall difference in accuracy was less than 0.1% using greater cell numbers (eTable 2 in the Supplement).

Friedewald-estimated LDL-C (LDL-C_F_) was calculated as total cholesterol−HDL-C−triglycerides/5. Although the Friedewald equation is not validated for triglyceride levels of 400 mg/dL or greater, we included it for comparison with prior literature.

The LDL-C estimated by the Sampson method (LDL-C_S_) was calculated using least-squares regressions, as described by Sampson et al^10^: LDL-C = (total cholesterol/0.948) − (HDL-C/0.971) − ([triglycerides/8.56] + [triglycerides × non–HDL-C/2140] − [triglycerides squared/16 100]) − 9.44.

The Sampson method was derived in a training data set of 4328 patients who were seen at the National Institutes of Health from 1976 to 1999 and had very high LDL-C and triglyceride levels.^10^ Multiple regression modeling for estimating β-quantification LDL-C was used to calculate coefficients.

### Statistical Analysis

All comparator analyses were conducted in the validation data set. Overall accuracy was defined as the proportion of direct LDL-C (dLDL-C) in the same category as estimated LDL-C based on the following estimated LDL-C levels: less than 40, 40 to 69, 70 to 99, 100 to 129, 130 to 159, 160 to 189, and 190 mg/dL or greater. We further examined accuracy of dLDL-C and estimated LDL-C for patients with estimated LDL-C levels less than 40 and less than 70 mg/dL based on triglyceride and non–HDL-C levels.

The magnitude of error was calculated as estimated LDL-C minus dLDL-C and the percentages of patients with error levels of less than 5, 5 to 9, 10 to 19, 20 to 29, and 30 mg/dL or more were calculated for each LDL-C estimation method, stratified by guideline LDL-C category. We also calculated the mean absolute difference between estimated and measured LDL-C. To understand the potential effects of fasting on study results, we performed a sensitivity analysis stratifying patients based on their fasting status. To convert cholesterol values from milligrams per deciliter to millimoles per liter, multiply by 0.0259. To convert triglyceride values from milligrams per deciliter to millimoles per liter, multiply by 0.0113. A 2-sided *P* < .05 was considered statistically significant. Statistical analyses were performed using Stata, version 15.1 (StataCorp LLC) and R, version 4.0.2 (R Foundation for Statistical Computing).

## Results

### Characteristics of Study Patients

A total of 111 939 patients (mean [SD] age, 52 [13] years; 65.0% male) with triglyceride levels of 400 to 799 mg/dL were included, representing 2.2% of 5 081 680 patients in the database. Data on race and ethnicity were not available because this is a clinical laboratory data set. Demographic and lipid characteristics of the derivation (n = 74 611) and validation (n = 37 328) data sets are summarized in Table 1. No significant differences were found between the data sets.

**Table 1.** Study Population Characteristics^a^

Characteristic	Overall (N = 111 939)	Derivation set (n = 74 611)	Validation set (n = 37 328)
Age, mean (SD), y	52 (13)	52 (13)	52 (13)
Age category, y			
<11	177 (0.2)	129 (0.2)	48 (0.1)
11-<18	537 (0.5)	341 (0.5)	196 (0.5)
≥18	110 355 (99.4)	73 576 (99.4)	36 779 (99.3)
Sex			
Female	38 884 (35.0)	25 914 (35.0)	12 970 (35.0)
Male	72 219 (65.0)	48 150 (65.0)	24 069 (65.0)
Fasting status			
Nonfasting	19 373 (54.7)	13 024 (54.6)	6349 (54.8)
Fasting	16 067 (45.3)	10 828 (45.4)	5239 (45.2)
Diabetes	10 101 (9.0)	6818 (9.1)	3283 (8.8)
Hypertension	7655 (6.8)	5213 (7.0)	2442 (6.5)
Lipid values, median (IQR), mg/dL			
Total cholesterol	219 (187-256)	219 (187-256)	220 (188-255)
HDL-C	35 (30-41)	35 (30-41)	35 (30-41)
LDL-C	114 (88-142)	114 (88-142)	114 (88-142)
Non–HDL-C	183 (153-217)	183 (153-217)	183 (153-217)
VLDL-C	66 (54-81)	66 (54-81)	66 (54-81)
Lp(a)-C	7 (5-12)	7 (5-12)	7 (5-12)
Triglycerides	484 (434-571)	484 (434-572)	485 (434-571)
Total cholesterol–VLDL-C ratio	3.3 (2.7-3.9)	3.3 (2.7-3.9)	3.3 (2.7-3.9)
Triglyceride–VLDL-C ratio	7.5 (6.3-9.0)	7.5 (6.3-9.0)	7.6 (6.3-9.0)
Triglyceride–total cholesterol ratio	2.3 (1.9-2.8)	2.3 (1.9-2.8)	2.3 (1.9-2.8)

Abbreviations: HDL-C, high-density lipoprotein cholesterol; LDL-C, low-density lipoprotein cholesterol; Lp(a)-C, lipoprotein(a) cholesterol; VLDL-C, very low-density lipoprotein cholesterol.

SI conversion factors: To convert cholesterol levels to millimoles per liter, multiply by 0.0259; to convert triglyceride levels to millimoles per liter, multiply by 0.0113.

^a^ Data are presented as number (percentage) of patients unless otherwise indicated.

### Distribution in Ratio of Triglycerides to VLDL-C

eFigure 1 in the Supplement illustrates the distribution of ratios of triglycerides to VLDL-C compared with triglyceride and non–HDL-C values. The median ratio of triglycerides to VLDL-C was 7.5 (IQR, 6.3-9.0); the fifth percentile for the ratio of triglycerides to VLDL-C was 4.8 and the 95th percentile was 12.3. Median ratios of triglycerides to VLDL-C along with their IQRs for 240, 560, and 1040 cell counts are provided in eTable 1 in the Supplement. eFigure 3 in the Supplement illustrates VLDL-C by triglycerides and non–HDL-C strata. eTables 4 and 5 in the Supplement give the medians and relative differences between estimated and direct VLDL-C, respectively.

### Overall Accuracy of LDL-C Estimation

**Figure 1.**
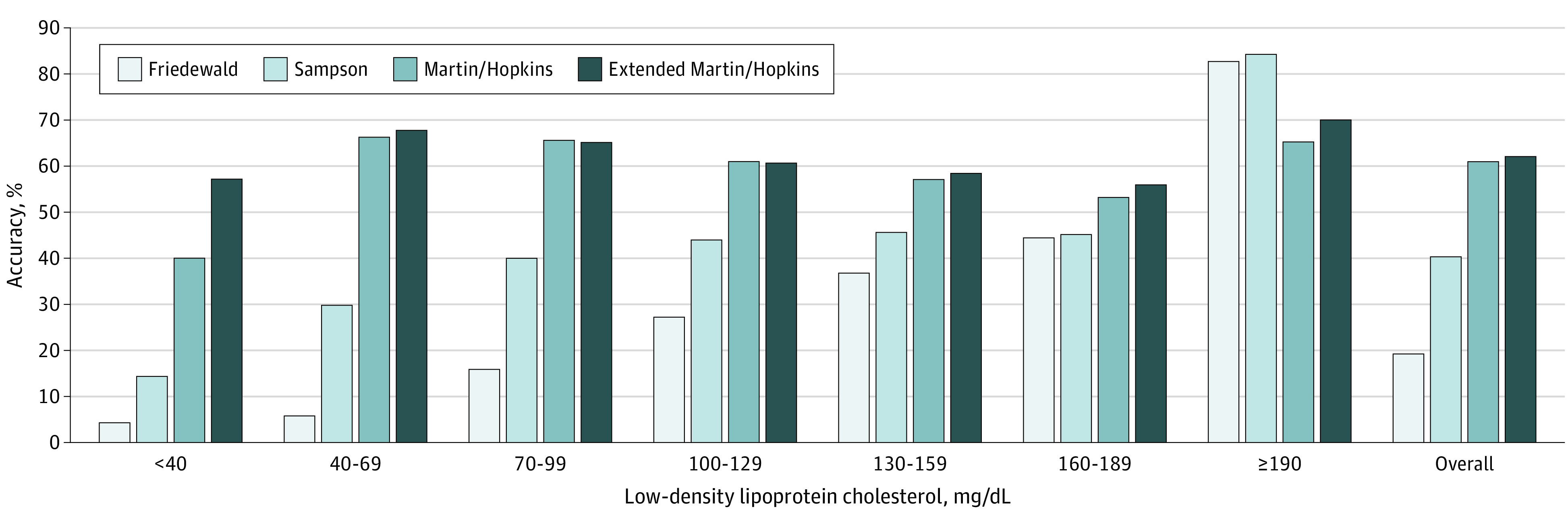
Accuracy in Guideline Classification by Various Methods in Relation to Direct Low-Density Lipoprotein Cholesterol for Hypertriglyceridemia SI conversion factor: To convert low-density lipoprotein cholesterol levels to millimoles per liter, multiply by 0.0259.

**Figure 2.**
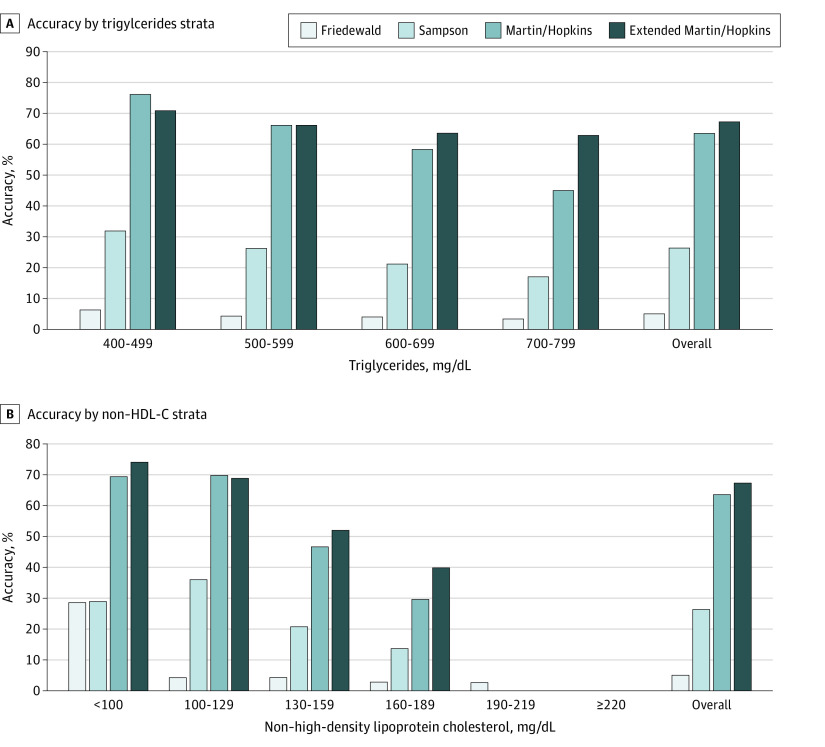
Accuracy Between Methods in Classifying Low-Density Lipoprotein Cholesterol Levels Lower Than 70 mg/dL SI conversion factor: To convert low-density lipoprotein cholesterol levels to millimoles per liter, multiply by 0.0259; to convert triglyceride levels to millimoles per liter, multiply by 0.0113. HDL-C indicates high-density lipoprotein cholesterol.

Across all individual guideline LDL-C classes, estimation of LDL-C by the extended Martin/Hopkins equation was most accurate (62.1%), followed by the Sampson (40.4%) and Friedewald (19.3%) equations (Figure 1). Consistent with prior National Institutes of Health findings, performance decreased at higher triglyceride levels; for example, at triglyceride levels of 800 to 999 mg/dL, the extended Martin/Hopkins equation accuracy was 47.3%, followed by accuracies of 19.9% with the Sampson equation and 7.3% with the Friedewald equation, for all LDL-C classes. In classifying LDL-C levels less than 70 mg/dL and across triglyceride strata up to 799 mg/dL, the extended Martin/Hopkins equation was most accurate (67.3%) compared with the accuracies of the Friedewald equation (5.1%) and the Sampson equation (26.4%) (Figure 2A). A similar pattern was seen in classifying LDL-C levels less than 40 mg/dL across triglyceride strata (eFigure 2A in the Supplement), with the extended Martin/Hopkins equation being most accurate (57.2%), followed by the Sampson (14.4%) and Friedewald (4.3%) equations.

### Extent of Guideline Misclassification at Low LDL-C Levels

**Figure 3.**
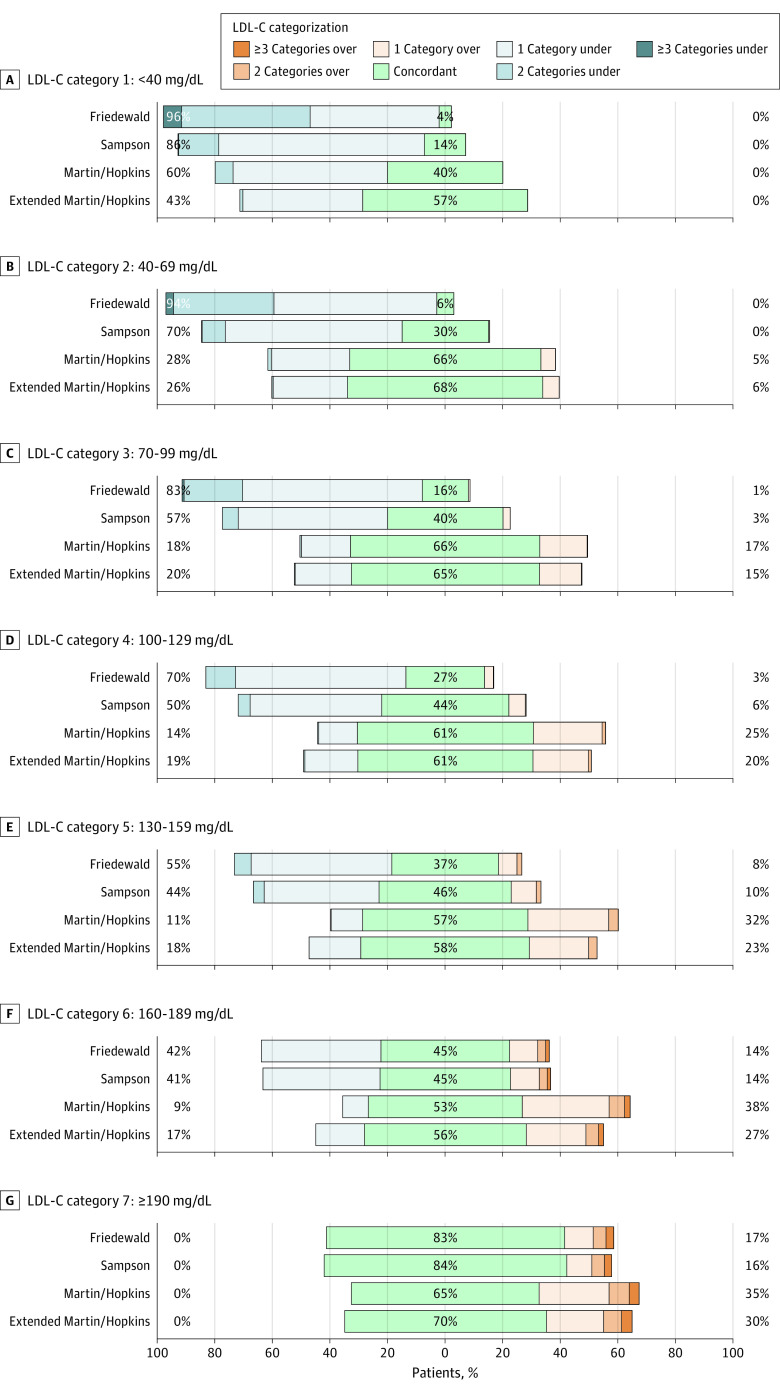
Proportion of Misclassified Patients per Direction by Estimated Low-Density Lipoprotein Cholesterol (LDL-C) Category Graphs represent the total percentage of patients who were underclassified and overclassified within each LDL-C category. Values to the left and right of 0 on the x-axis indicate percentage underclassified and percentage overclassified, respectively. SI conversion factor: To convert low-density lipoprotein cholesterol levels to millimoles per liter, multiply by 0.0259.

In classifying LDL-C levels less than 40 mg/dL, underestimation of LDL-C was present in 43% of individuals with the extended Martin/Hopkins equation compared with 86% with the Sampson equation and 96% with the Friedewald equation (Figure 3). The degree of underestimation was least pronounced with the extended Martin/Hopkins equation, with only 1.1% of patients underclassified by 2 guideline categories (ie, dLDL-C ≥70 mg/dL), compared with 14.1% with the Sampson equation and 51% with the Friedewald equation.

In classifying LDL-C levels of 40 to 69 mg/dL, underestimation of LDL-C occurred in 26% with the extended Martin/Hopkins equation compared with 70% with the Sampson equation and 94% with the Friedewald equation (Figure 3). The degree of underestimation was least pronounced with the extended Martin/Hopkins equation, with only 0.5% of patients underclassified by 2 categories (ie, dLDL-C >100 mg/dL), compared with 8.2% with the Sampson equation and 37.5% with the Friedewald equation.

### Magnitude of Patient-Level Error

Overall, the magnitude of error between the extended Martin/Hopkins equation LDL-C_E_ and dLDL-C was smaller compared with the Friedewald and Sampson equations (Table 2). In patients with LDL-C levels less than 40 mg/dL, 92.5% with LDL-C_F_ and 38.7% with LDL-C_S_ had differences of 30 mg/dL or greater between estimated LDL-C and dLDL-C compared with only 2.7% of patients who had differences of 30 mg/dL or greater between LDL-C_E_ and dLDL-C. Moreover, 0.1% with LDL-C_F_ and 2.8% with LDL-C_S_ had differences of less than 5 mg/dL between estimated LDL-C and dLDL-C compared with 32.6% of patients who had differences of 5 mg/dL or less between LDL-C_E_ and dLDL-C. eFigure 4 in the Supplement illustrates the median differences between estimated LDL-C and dLDL-C levels by triglyceride level across methods. At LDL-C levels of 40 to 69 mg/dL, 73.3% had differences of 30 mg/dL or greater with LDL-C_F_ and 23.0% with LDL-C_S_ between estimated LDL-C and dLDL-C compared with 2.3% of patients who had differences of 30 mg/dL or greater between LDL-C_E_ and dLDL-C.

**Table 2.** Percentage of Patients by Absolute Magnitude of Error Between Estimated LDL-C and Direct LDL-C for Triglyceride Levels of 400 to 799 mg/dL

Equation	LDL-C error				
	<5 mg/dL	5-9 mg/dL	10-19 mg/dL	20-29 mg/dL	≥30 mg/dL
**LDL-C: <40 mg/dL**					
Friedewald	0.1 (0.0-0.2)	0.1 (0.0-0.2)	1.3 (1.1-1.6)	6.0 (5.5-6.6)	92.5 (91.9-93.1)
Sampson	2.8 (2.2-3.7)	4.4 (3.6-5.4)	22.6 (20.8-24.5)	31.4 (29.4-33.5)	38.7 (36.6-40.9)
Martin/Hopkins	20.3 (16.4-24.9)	22.6 (18.5-27.3)	27.2 (22.8-32.1)	17.8 (14.1-22.2)	12.0 (9.0-15.9)
Extended^a^	32.6 (26.2-39.7)	30.5 (24.3-37.5)	25.7 (19.9-32.5)	8.6 (5.3-13.5)	2.7 (1.1-6.3)
**LDL-C: 40-69 mg/dL**					
Friedewald	1.0 (0.8-1.3)	1.5 (1.3-1.8)	7.2 (6.6-7.8)	16.9 (16.1-17.7)	73.3 (72.4-74.3)
Sampson	8.8 (8.2-9.5)	11.4 (10.6-12.2)	29.5 (28.5-30.6)	27.2 (26.1-28.3)	23.0 (22.0-24.1)
Martin/Hopkins	31.1 (29.5-32.8)	25.2 (23.7-26.8)	29.2 (27.6-30.8)	9.7 (8.7-10.9)	4.8 (4.1-5.6)
Extended^a^	32.9 (31.3-34.6)	26.3 (24.8-27.9)	30.2 (28.6-31.8)	8.4 (7.5-9.4)	2.3 (1.8-2.9)
**LDL-C: 70-99 mg/dL**					
Friedewald	2.9 (2.6-3.3)	4.4 (4.0-4.8)	13.8 (13.1-14.6)	24.2 (23.3-25.1)	54.6 (53.6-55.6)
Sampson	12.7 (12.1-13.4)	12.8 (12.2-13.4)	30.4 (29.5-31.3)	25.4 (24.6-26.2)	18.7 (18.0-19.5)
Martin/Hopkins	29.8 (28.9-30.8)	24.1 (23.2-25.1)	30.7 (29.7-31.7)	11.3 (10.7-12.0)	4.0 (3.6-4.5)
Extended^a^	29.9 (29.0-30.8)	25.2 (24.3-26.1)	31.3 (30.3-32.2)	10.2 (9.6-10.8)	3.5 (3.1-3.9)
**LDL-C: 100-129 mg/dL**					
Friedewald	6.6 (6.0-7.2)	7.6 (7.0-8.3)	20.7 (19.7-21.7)	25.8 (24.7-26.8)	39.4 (38.2-40.5)
Sampson	12.8 (12.2-13.5)	13.7 (13.0-14.4)	30.0 (29.1-30.9)	24.9 (24.0-25.7)	18.6 (17.9-19.4)
Martin/Hopkins	27.9 (27.1-28.7)	23.8 (23.0-24.6)	29.5 (28.7-30.4)	12.4 (11.8-13.1)	6.4 (6.0-6.9)
Extended^a^	27.7 (26.9-28.5)	24.3 (23.5-25.1)	30.1 (29.3-30.9)	11.9 (11.4-12.5)	6.0 (5.6-6.4)
**LDL-C: 130-159 mg/dL**					
Friedewald	9.6 (8.7-10.5)	11.1 (10.1-12.1)	25.2 (23.8-26.6)	25.3 (23.9-26.7)	28.9 (27.5-30.4)
Sampson	13.8 (12.9-14.8)	14.1 (13.1-15.1)	29.6 (28.4-30.9)	23.0 (21.8-24.1)	19.6 (18.5-20.7)
Martin/Hopkins	26.2 (25.2-27.1)	21.3 (20.5-22.2)	29.8 (28.8-30.8)	13.1 (12.4-13.9)	9.6 (9.0-10.2)
Extended^a^	24.6 (23.7-25.6)	22.0 (21.1-22.9)	30.8 (29.8-31.8)	13.7 (13.0-14.5)	8.9 (8.3-9.6)
**LDL-C: 160-189 mg/dL**					
Friedewald	12.8 (11.3-14.4)	12.7 (11.2-14.3)	28.7 (26.7-30.9)	23.3 (21.4-25.4)	22.5 (20.6-24.5)
Sampson	12.8 (11.5-14.3)	14.2 (12.8-15.8)	27.7 (25.8-29.6)	22.6 (20.9-24.5)	22.6 (20.9-24.4)
Martin/Hopkins	22.8 (21.4-24.2)	21.1 (19.8-22.4)	29.0 (27.5-30.5)	13.2 (12.1-14.3)	14.0 (12.9-15.1)
Extended^a^	22.6 (21.1-24.1)	20.5 (19.0-22.0)	29.8 (28.2-31.5)	13.8 (12.6-15.1)	13.3 (12.1-14.6)
**LDL-C: ≥190 mg/dL**					
Friedewald	17.0 (15.0-19.2)	15.7 (13.8-17.8)	25.6 (23.3-28.0)	17.9 (15.9-20.1)	23.8 (21.5-26.2)
Sampson	12.6 (10.8-14.7)	12.8 (11.0-14.9)	24.8 (22.4-27.3)	20.0 (17.8-22.4)	29.8 (27.2-32.4)
Martin/Hopkins	17.0 (15.6-18.4)	15.6 (14.3-17.1)	23.7 (22.1-25.4)	14.8 (13.5-16.3)	28.9 (27.1-30.6)
Extended^a^	18.4 (16.8-20.1)	17.2 (15.7-18.9)	25.6 (23.8-27.4)	14.1 (12.7-15.7)	24.7 (22.9-26.5)

Abbreviation: LDL-C, low-density lipoprotein cholesterol.

SI conversion factor: To convert LDL-C to millimoles per liter, multiply by 0.0259.

^a^ Six non–high-density lipoprotein cholesterol categories.

### Stratification by Fasting Status

Accuracy for LDL-C estimation across the Friedewald (25.2% vs 15.9%, *P* < .001) and Sampson (49.6% vs 35.0%, *P* < .001) equations was sensitive to fasting status, whereas the extended Martin/Hopkins equation did not differ in accuracy between fasting vs nonfasting patients (61.4% vs 62.4%, *P* = .27).

## Discussion

In this cross-sectional study, there were several important, novel findings: (1) across all LDL-C classes in hypertriglyceridemia (triglyceride levels of 400-799 mg/dL), LDL-C levels determined by the extended Martin/Hopkins equation were more accurate (62.1%) compared with the Friedewald (19.3%) and Sampson (40.4%) equations, (2) in classifying LDL-C levels of less than 70 and less than 40 mg/dL, the extended Martin/Hopkins equation was most accurate compared with the Friedewald and Sampson equations, (3) the magnitude of error between the extended Martin/Hopkins equation and dLDL-C was smaller, especially at low LDL-C levels, compared with the Friedewald and Sampson equations, and (4) there was considerable underclassification of LDL-C at low levels across all methods, but particularly with the Friedewald and Sampson equations, raising concern for undertreatment. Collectively, these findings provide insight into the extent of accuracy of LDL-C estimation across methods at high triglyceride levels.

### LDL-C Estimation

Several other methods of LDL-C estimation have been previously described and not well validated.^11,12,13^ The Martin/Hopkins and Friedewald equations were validated for patients with serum triglyceride levels less than 400 mg/dL because at higher triglyceride levels chylomicrons may alter the association between triglycerides and VLDL-C; however, in fasting samples, chylomicronemia is rare, and the extent to which this is a complication for nonfasting samples is not well defined.^5,14,15^ Furthermore, the Martin/Hopkins equation has demonstrated little difference between fasting and nonfasting samples. A previous study^5^ found that the Martin/Hopkins equation improved LDL-C accuracy compared with Friedewald estimation and was most clinically useful in patients with moderate hypertriglyceridemia (triglyceride levels of 150-399 mg/dL) and low LDL-C levels. Large laboratories and studies across the world have adopted and validated this equation.^16,17,18,19^ Moreover, the 2018 AHA/ACC Cholesterol Guideline provided a class IIa recommendation for use of the Martin/Hopkins equation in patients with LDL-C levels less than 70 mg/dL and a class IIa recommendation for direct cholesterol measurement. European guidelines have also recommended the Martin/Hopkins equation for calculating LDL-C levels.^1,20^ The Martin/Hopkins calculator is available online.^21^

Recently, the Sampson equation was introduced, with a focus on triglyceride levels of 400 mg/dL or greater. The equation was derived in a population that was much smaller (training data set of 4328 patients) and included samples from the 1970s to 1990s with highly skewed lipid levels (LDL-C levels of 200-800 mg/dL and triglyceride levels of ≥2880 mg/dL), which are not reflective of patients seen in routine practice compared with the Martin/Hopkins equation that was derived in a contemporary cohort with more than 1 million lipid samples from patients seen in routine practice, with a lipid distribution shown to be analogous to the National Health and Nutrition Examination Survey, a nationally representative cohort.^8^

Interest in conducting lipid assessment in the nonfasting state is increasing. The Sampson equation has a significant difference in accuracy for LDL-C estimation in fasting vs nonfasting patients (49.6% vs 35.0%, *P* < .001). The extended Martin/Hopkins equation, on the other hand, has no significant difference in overall accuracy in fasting vs nonfasting patients (61.4% vs 62.4%, *P* = .27), thereby better supporting routine clinical scenarios in which patients are generally not fasting.^22,23^

### Underestimation at Low LDL-C Levels

Low-density lipoprotein cholesterol remains an important treatment target, with emphasis in worldwide guidelines that lower is better.^1,4,24^ The 2018 AHA/ACC Cholesterol Guideline recommends an LDL-C threshold of 70 mg/dL or greater in patients with atherosclerotic cardiovascular disease to consider adding nonstatin therapy to high-intensity statin therapy, and the European Society of Cardiology recommends an LDL-C goal of less than 40 mg/dL in very high-risk patients with recurrent events.^1,4,19,25^ In this study, we found that at LDL-C levels of less than 70 mg/dL and less than 40 mg/dL, the extended Martin/Hopkins equation was most accurate compared with the Friedewald and Sampson equations, which have a tendency to underestimate LDL-C levels. This underestimation could lead to false reassurance about LDL-C and missed opportunities for prevention.

The method of statistical analysis may explain some of these differences in our findings from the Sampson equation publication.^10^ In the Sampson equation publication,^10^ LDL-C was first classified by direct (β quantification) LDL-C categories, which functions to suppress the ability to detect underestimation. Statistically, this is problematic because in the group with the lowest LDL-C levels, in which most underestimation is anticipated to occur, underestimation cannot be detected because the lowest category is made up entirely of individuals based on a directly measured LDL-C. This method also represents a less ideal approach from a practical clinical perspective because the practitioner and patient receive an estimated LDL-C value from a standard lipid profile in real-world practice. The relevant question facing a practitioner and patient is whether a measured result is likely to agree with the laboratory-estimated LDL-C value, reflecting the approach that we have used in this study. Lastly, although LDL-C remains an important treatment target, other broader measures of atherogenic lipoprotein burden, such as non–HDL-C and apolipoprotein B, may be considered in hypertriglyceridemia according to recent guidelines.^1,26^

### Implications for Patient Care

These findings are important because practitioners and patients may forgo initiation or titration of atherosclerotic cardiovascular disease risk-reducing therapy or decide to discontinue or reduce lipid-lowering therapies by incorrectly believing the LDL-C value is well controlled when an underestimated value is reported, resulting in undertreatment of high-risk patients and a lost opportunity for prevention. Despite higher accuracy with the extended Martin/Hopkins equation, it could be argued that all 3 equations are considerably inaccurate at triglyceride levels of 400 mg/dL or greater. However, the Friedewald and Sampson equations had more underestimation at low LDL-C levels (<70 mg/dL) compared with the Martin/Hopkins equation, thus leading to greater undertreatment in high-risk patients. Nearly all (96% using the Friedewald equation and 86% using the Sampson equation) actually had LDL-C values of 40 mg/dL or greater when classified as less than 40 mg/dL. These trends of marked underclassification with the Friedewald and Sampson equations remained at higher LDL-C levels, in effect missing patients with severely elevated LDL-C levels and familial hypercholesterolemia. For instance, in the LDL-C category of 130 to 159 mg/dL (Figure 3), on the basis of the Friedewald and Sampson equations, more patients were underclassified by 1 to 2 categories compared with the extended Martin/Hopkins equation. A similar trend was observed at LDL-C levels of 160 to 189 mg/dL with the Friedewald and Sampson equations. Clinically, these are missed cases of LDL-C levels of 190 mg/dL or greater and potential familial hypercholesterolemia diagnoses.

In addition to misclassification, our study documents a large magnitude of error, which corroborates the large mean absolute difference in LDL-C of 24.9 mg/dL previously reported for the Sampson equation. Notably, 18.7% of patients using the Sampson equation and 54.6% using the Friedewald equation had a 30-mg/dL or higher error between estimated LDL-C and dLDL-C at levels of 70 to 99 mg/dL (compared with 3.5% using the extended Martin/Hopkins equation). An error of 30 mg/dL represents 1 full stratum in guideline LDL-C categories and therefore represents a large error from a clinical perspective. This error can influence clinical decision-making by reclassifying patients incorrectly and leading to undertreatment.^27^

Overall, the error in LDL-C estimation is consistent with the known problem of chylomicrons distorting LDL-C estimation at triglyceride levels of 400 mg/dL or higher.^15^ The immediate clinical priority in these individuals is triglyceride reduction to prevent pancreatitis. With more aggressive lowering of triglyceride levels through lifestyle modification and pharmaceutical therapy, LDL-C may be more accurately estimated during follow-up. However, whether such errors at high triglyceride levels should be acceptable needs to be explored further, given its implications in management of high- and very high-risk patients.^28^ In these instances, dLDL-C testing may be considered, as currently endorsed by the AHA/ACC Cholesterol Guideline (class IIa recommendation), and further studies are needed on dLDL-C testing.

### Limitations

This study has some limitations. Although lipid distributions in our study closely matched a nationally representative population-based survey, patients who had cholesterol concentrations quantified by vertical spin density gradient ultracentrifugation may represent a different population. Race and ethnicity, obesity, insulin resistance, and lipid treatment were not available for analysis. Genetic factors may play a major role in shaping the metabolic state of an individual and may vary among racial and ethnic groups regarding the kind of dyslipidemia they may have. This information was not available for the current analysis. We also used the first available lipid sample for patients, thereby not addressing intraindividual variation between subsequent samples that affect LDL-C classification.

Concern has also been raised, based on limited data (inconclusive analysis of a small sample presented briefly in a review article^15^), that the VAP ultracentrifugation method might report falsely low VLDL-C values in samples with high triglyceride levels because of adherence of triglyceride-rich lipoproteins to centrifuge tube walls.^10,15,29,30^ However, this concern is not unique to the VAP method of ultracentrifugation and likely is only of importance at triglyceride levels beyond the range in this analysis (in which case LDL-C is not typically reported in practice). Furthermore, it would not impact LDL-C measurement because LDL separates in the middle of the centrifuge tube, well away from where VLDL and chylomicrons peak. The VAP method has undergone random split-sample validation compared with the β-quantification ultracentrifugation LDL-C values from the Washington University in St. Louis laboratory.^9^

## Conclusions

Using a large, nationally representative patient sample, this study assessed accuracy of LDL-C estimation in patients with triglyceride levels beyond the traditional boundary. The extended Martin/Hopkins equation provided more accurate estimation than the Friedewald and Sampson equations. However, the extent of inaccuracy was still notable across methods, particularly at LDL-C levels less than 40 mg/dL. Accuracy of LDL-C estimation by the extended Martin/Hopkins equation is unaffected with respect to fasting status compared with the Friedewald and Sampson equations, which had larger inaccuracy in nonfasting samples. The results of this study suggest that, overall, the extended Martin/Hopkins equation provides the best available estimation of LDL-C in patients with triglyceride levels of 400 to 799 mg/dL and indicates a need for change in the use of Friedewald and Sampson equations.

## Notes

Supplement. **eTable 1.** Median and IQR for TG:VLDL-C by Non-HDL-C and Triglyceride Strata (240-Cell, 560-Cell, and 1040-Cell) **eTable 2.** Accuracy in LDL-C Classification by Extended Martin/Hopkins Method by Various Cell Counts **eTable 3.** Percentage of Patients by Absolute Error Between Estimated LDL-C and Direct LDL-C for TG 400-799 mg/dL **eTable 4.** Median Difference Between Estimated and Direct VLDL-C by TG and Non-HDL-C Strata **eTable 5.** Relative Difference Between Estimated and Direct VLDL-C by TG and Non-HDL-C Strata **eFigure 1.** TG:VLDL-C Ratio by Triglyceride and Non-HDL-C Strata **eFigure 2.** Accuracy Between Methods in LDL-C Classification at <40 mg/dL by TG and Non-HDL-C Strata **eFigure 3.** VLDL-C by Triglyceride and Non-HDL-C Strata **eFigure 4.** Median Difference Between Estimated and Direct LDL-C Levels by TG Level
