# Supplementary material for: Comparison of Methods to Estimate Low-Density Lipoprotein Cholesterol in Patients With High Triglyceride Levels
Source: JAMA Netw Open. 2021 Oct 28;4(10):e2128817. doi: 10.1001/jamanetworkopen.2021.28817 (PMC8554644; doi:10.1001/jamanetworkopen.2021.28817)
Supplement: Supplement. — eTable 1. Median and IQR for TG:VLDL-C by Non-HDL-C and Triglyceride Strata (240-Cell, 560-Cell, and 1040-Cell) eTable 2. Accuracy in LDL-C Classification by Extended Martin/Hopkins Method by Various Cell Counts eTable 3. Percentage of Patients by Absolute Error Between Estimated LDL-C and Direct LDL-C for TG 400-799 mg/dL eTable 4. Median Difference Between Estimated and Direct VLDL-C by TG and Non-HDL-C Strata eTable 5. Relative Difference Between Estimated and Direct VLDL-C by TG and Non-HDL-C Strata eFigure 1. TG:VLDL-C Ratio by Triglyceride and Non-HDL-C Strata eFigure 2. Accuracy Between Methods in LDL-C Classification at <40 mg/dL by TG and Non-HDL-C Strata eFigure 3. VLDL-C by Triglyceride and Non-HDL-C Strata eFigure 4. Median Difference Between Estimated and Direct LDL-C Levels by TG Level [file jamanetwopen-e2128817-s001.pdf]

Supplementary Online Content

Sajja A, Park J, Sathiyakumar V, et al. Comparison of methods to estimate low-density lipoprotein cholesterol in patients with high triglyceride levels. *JAMA Netw Open*. 2021;4(10):e2128817. doi:10.1001/jamanetworkopen.2021.28817

eTable 1. Median and IQR for TG:VLDL-C by Non-HDL-C and Triglyceride Strata (240-Cell, 560-Cell, and 1040-Cell)

eTable 2. Accuracy in LDL-C Classification by Extended Martin/Hopkins Method by Various Cell Counts

eTable 3. Percentage of Patients by Absolute Error Between Estimated LDL-C and Direct LDL-C for TG 400-799 mg/dL

eTable 4. Median Difference Between Estimated and Direct VLDL-C by TG and Non-HDL-C Strata

eTable 5. Relative Difference Between Estimated and Direct VLDL-C by TG and Non-HDL-C Strata

eFigure 1. TG:VLDL-C Ratio by Triglyceride and Non-HDL-C Strata

eFigure 2. Accuracy Between Methods in LDL-C Classification at <40 mg/dL by TG and Non-HDL-C Strata

eFigure 3. VLDL-C by Triglyceride and Non-HDL-C Strata

eFigure 4. Median Difference Between Estimated and Direct LDL-C Levels by TG Level

This supplementary material has been provided by the authors to give readers additional information about their work.

© 2021 Sajja A et al. *JAMA Network Open.*

eTable 1. Median and IQR for TG:VLDL-C by Non-HDL-C and Triglyceride Strata

A. 240-Cell

TG level,	Non-HDL-C, mg/dL					
mg/dL	<100	100-129	130-159	160-189	190-219	220+
400-409	10.4 (9.1-12.5)	8.7 (7.6-10.2)	7.9 (6.9-9.1)	7.3 (6.4-8.4)	6.7 (5.9-7.7)	6.1 (5.1-6.9)
410-419	10.7 (9.4-12.7)	8.9 (7.7-10.2)	7.9 (6.9-9.2)	7.3 (6.4-8.4)	6.7 (5.9-7.8)	6.0 (5.1-7.0)
420-429	10.3 (9.2-13.7)	8.9 (7.8-10.4)	7.9 (6.9-9.2)	7.4 (6.3-8.4)	6.8 (5.9-7.7)	6.0 (5.1-7.0)
430-439	11.2 (9.2-14.0)	8.9 (7.8-10.8)	8.0 (7.0-9.3)	7.3 (6.4-8.4)	6.8 (5.9-7.7)	6.0 (5.1-6.9)
440-449	12.0 (10.3-13.9)	9.0 (8.0-10.5)	8.0 (7.0-9.3)	7.5 (6.3-8.6)	6.9 (5.9-7.9)	6.0 (5.1-7.1)
450-459	11.3 (9.6-13.4)	9.3 (8.1-11.0)	8.2 (7.1-9.3)	7.4 (6.4-8.6)	7.0 (6.1-8.1)	6.0 (5.2-7.0)
460-469	12.3 (10.2-14.2)	9.2 (8.0-10.9)	8.3 (7.2-9.6)	7.7 (6.5-8.8)	6.9 (5.9-7.9)	6.1 (5.1-7.1)
470-479	10.6 (9.3-13.1)	9.3 (8.2-11.0)	8.3 (7.2-9.7)	7.6 (6.6-8.9)	7.0 (6.1-8.1)	6.0 (5.1-7.1)
480-489	11.7 (10.1-15.6)	9.3 (8.3-10.9)	8.4 (7.3-9.8)	7.6 (6.7-8.9)	7.1 (6.1-8.2)	6.1 (5.2-7.2)
490-499	11.6 (10.7-13.2)	9.6 (8.4-11.4)	8.4 (7.4-9.6)	7.6 (6.7-8.8)	7.2 (6.1-8.3)	6.2 (5.2-7.2)
500-509	12.1 (10.3-14.8)	9.2 (8.2-10.8)	8.4 (7.4-10.0)	7.5 (6.6-8.8)	7.1 (6.1-8.2)	6.2 (5.2-7.2)
510-519	12.3 (10.7-14.0)	9.9 (8.6-11.6)	8.5 (7.3-10.0)	7.9 (6.9-9.1)	7.1 (6.1-8.2)	6.3 (5.4-7.3)
520-529	12.0 (10.3-15.9)	9.8 (8.6-12.0)	8.7 (7.6-10.2)	7.7 (6.7-8.9)	7.1 (6.3-8.3)	6.3 (5.3-7.3)
530-539	12.0 (9.8-14.8)	9.8 (8.7-11.4)	8.7 (7.5-10.2)	7.8 (6.7-9.3)	7.2 (6.2-8.5)	6.3 (5.3-7.3)
540-549	11.3 (9.8-14.4)	10.0 (9.0-12.2)	8.8 (7.7-10.1)	7.8 (6.8-9.1)	7.4 (6.4-8.6)	6.3 (5.4-7.4)
550-559	12.2 (10.4-15.9)	10.2 (8.9-11.8)	8.8 (7.7-10.5)	8.0 (6.9-9.2)	7.4 (6.4-8.4)	6.2 (5.2-7.3)
560-569	13.8 (11.3-17.2)	10.2 (8.9-12.5)	8.7 (7.6-10.2)	8.1 (7.0-9.2)	7.2 (6.2-8.2)	6.2 (5.3-7.3)
570-579	15.4 (11.6-19.9)	10.4 (9.0-12.2)	8.9 (7.7-10.6)	8.0 (6.9-9.4)	7.3 (6.4-8.2)	6.2 (5.2-7.3)
580-589	12.7 (11.6-17.7)	10.5 (9.0-12.2)	9.1 (7.8-10.6)	8.3 (7.2-9.4)	7.3 (6.2-8.5)	6.4 (5.4-7.6)
590-599	12.5 (10.8-19.3)	10.5 (9.1-11.7)	9.2 (7.9-11.3)	8.3 (7.2-9.9)	7.2 (6.5-8.7)	5.9 (5.1-7.4)
600-609	13.7 (11.8-22.3)	10.5 (9.2-13.2)	8.9 (7.8-10.4)	8.2 (7.1-9.3)	7.6 (6.5-8.6)	6.3 (5.3-7.3)
610-619	15.4 (11.6-18.1)	10.5 (9.1-13.4)	9.1 (8.1-10.4)	8.4 (7.1-10.0)	7.5 (6.4-8.7)	6.4 (5.4-7.5)
620-629	16.4 (12.2-22.5)	11.3 (9.4-14.1)	9.2 (8.1-10.4)	8.5 (7.2-10.2)	7.5 (6.4-8.8)	6.4 (5.3-7.6)
630-639	14.1 (12.2-16.4)	11.6 (9.8-14.2)	9.4 (8.1-11.2)	8.2 (7.4-9.4)	7.3 (6.3-8.6)	6.2 (5.4-7.2)
640-649	14.8 (12.9-16.9)	11.0 (9.6-12.7)	9.1 (7.8-10.7)	8.1 (7.0-9.7)	7.5 (6.5-8.4)	6.6 (5.6-7.2)
650-659	14.2 (11.9-23.3)	11.0 (9.6-13.4)	9.2 (8.2-11.1)	8.3 (7.3-10.1)	7.5 (6.6-8.8)	6.4 (5.4-7.7)
660-669	15.0 (12.2-18.9)	10.9 (9.6-13.0)	9.2 (7.8-11.0)	8.3 (7.3-9.5)	7.5 (6.8-8.6)	6.5 (5.6-7.7)
670-679	14.2 (12.1-20.4)	11.0 (9.8-12.9)	9.3 (8.3-10.6)	8.6 (7.4-9.8)	7.6 (6.7-8.9)	6.7 (5.4-7.8)
680-689	16.7 (13.5-19.8)	11.5 (10.2-13.5)	9.8 (8.6-12.0)	8.3 (7.3-9.6)	7.4 (6.6-8.6)	6.7 (5.7-7.9)
690-699	15.0 (12.2-18.4)	11.6 (9.8-13.9)	9.8 (8.5-12.1)	8.4 (7.2-10.0)	7.8 (6.6-9.0)	6.5 (5.5-7.5)
700-709	16.6 (13.1-20.2)	11.5 (9.5-13.7)	9.5 (8.5-10.8)	8.5 (7.2-10.0)	7.8 (6.6-9.3)	6.9 (5.7-7.8)

© 2021 Sajja A et al. *JAMA Network Open.*

710-719	14.5 (11.1-19.3)	10.9 (9.8-12.9)	9.7 (8.6-10.7)	8.5 (7.4-9.7)	7.8 (6.5-9.0)	6.4 (5.6-7.7)
720-729	16.5 (13.5-26.8)	11.7 (9.7-14.6)	9.5 (8.5-10.9)	8.5 (7.7-10.0)	7.6 (6.9-9.1)	6.6 (5.6-7.8)
730-739	18.2 (15.4-22.2)	12.2 (10.8-15.2)	9.9 (8.9-11.5)	8.9 (7.8-10.4)	8.2 (6.9-9.4)	6.6 (5.6-7.7)
740-749	17.5 (14.2-19.0)	11.7 (10.4-14.6)	9.9 (8.6-11.8)	8.5 (7.6-10.1)	7.9 (6.8-9.2)	6.6 (5.4-7.5)
750-759	17.5 (15.8-21.6)	12.9 (11.0-15.7)	10.2 (8.5-12.6)	8.8 (7.6-10.2)	8.1 (7.0-9.5)	6.4 (5.2-7.6)
760-769	19.2 (13.1-21.2)	11.4 (10.2-13.7)	9.9 (9.0-12.1)	8.7 (7.6-10.3)	8.3 (7.2-9.6)	6.5 (5.6-7.6)
770-779	17.3 (12.3-24.2)	13.4 (11.2-15.2)	10.4 (9.1-12.3)	8.6 (7.7-10.8)	8.2 (6.9-9.3)	6.7 (5.8-8.1)
780-789	23.9 (16.4-27.0)	12.3 (10.7-13.7)	10.4 (9.3-11.8)	9.1 (7.8-10.1)	7.9 (6.9-9.1)	6.7 (5.4-7.8)
790-799	15.6 (13.9-20.3)	13.0 (12.1-19.4)	10.7 (9.1-13.4)	8.7 (7.9-9.7)	8.0 (7.0-9.0)	6.7 (5.8-7.8)

© 2021 Sajja A et al. *JAMA Network Open.*

B. 560-Cell

TG level, mg/dL	Non-HDL-C, mg/dL													
	<100	100-109	110-119	120-129	130-139	140-149	150-159	160-169	170-179	180-189	190-199	200-209	210-219	220+
400-409	10.4 (9.1-12.5)	9.0 (8.0-10.8)	8.9 (7.7-10.3)	8.5 (7.4-9.6)	8.3 (7.2-9.5)	7.8 (6.7-9.0)	7.7 (6.6-8.8)	7.4 (6.5-8.5)	7.4 (6.4-8.4)	7.1 (6.2-8.1)	6.9 (6.1-7.8)	6.7 (5.9-7.6)	6.6 (5.8-7.7)	6.1 (5.1-6.9)
410-419	10.7 (9.4-12.7)	9.3 (8.3-10.6)	9.1 (7.9-10.6)	8.5 (7.5-9.8)	8.3 (7.4-9.8)	7.9 (6.9-9.1)	7.6 (6.6-8.8)	7.5 (6.6-8.7)	7.4 (6.4-8.4)	7.1 (6.2-8.2)	6.9 (6.1-7.9)	6.7 (5.8-7.9)	6.5 (5.7-7.5)	6.0 (5.1-7.0)
420-429	10.3 (9.2-13.7)	9.0 (7.8-10.6)	9.0 (8.1-10.9)	8.8 (7.6-10.0)	8.4 (7.0-9.6)	7.9 (7.0-9.0)	7.7 (6.7-9.1)	7.7 (6.7-8.5)	7.4 (6.3-8.5)	7.0 (6.1-8.2)	7.0 (6.0-7.8)	6.9 (6.0-7.9)	6.6 (5.6-7.5)	6.0 (5.1-7.0)
430-439	11.2 (9.2-14.0)	9.1 (8.0-11.2)	9.2 (8.0-11.1)	8.8 (7.6-10.1)	8.3 (7.3-9.5)	8.0 (7.0-9.4)	7.8 (6.7-9.0)	7.6 (6.5-8.8)	7.3 (6.3-8.4)	7.2 (6.4-8.3)	7.0 (6.1-8.0)	6.8 (5.8-7.6)	6.5 (5.6-7.6)	6.0 (5.1-6.9)
440-449	12.0 (10.3-13.9)	9.2 (8.5-10.9)	9.2 (8.1-10.5)	8.7 (7.5-10.2)	8.1 (7.1-9.4)	8.3 (7.3-9.5)	7.9 (6.8-9.1)	7.6 (6.4-8.8)	7.4 (6.3-8.5)	7.4 (6.3-8.5)	7.0 (6.0-8.1)	6.9 (6.0-7.8)	6.6 (5.6-7.5)	6.0 (5.1-7.1)
450-459	11.3 (9.6-13.4)	10.0 (8.4-11.9)	9.4 (7.9-10.8)	9.0 (7.9-10.6)	8.4 (7.3-9.7)	8.1 (7.1-9.3)	8.1 (6.9-9.0)	7.8 (6.6-8.9)	7.2 (6.3-8.4)	7.2 (6.2-8.5)	7.2 (6.3-8.3)	7.0 (6.2-7.9)	6.8 (6.0-7.7)	6.0 (5.2-7.0)
460-469	12.3 (10.2-14.2)	9.9 (8.5-11.0)	9.2 (7.9-10.9)	8.9 (7.8-10.8)	8.5 (7.4-10.2)	8.4 (7.2-9.7)	8.0 (7.2-9.1)	7.8 (6.7-9.0)	7.8 (6.6-8.9)	7.5 (6.5-8.6)	7.0 (6.1-8.1)	6.9 (5.9-7.9)	6.7 (5.6-7.6)	6.1 (5.1-7.1)
470-479	10.6 (9.3-13.1)	10.1 (9.0-11.7)	9.1 (7.9-10.6)	9.0 (8.1-10.6)	8.6 (7.5-9.9)	8.3 (7.2-10.0)	8.1 (7.1-9.5)	7.8 (6.8-9.2)	7.7 (6.8-9.0)	7.2 (6.2-8.5)	7.2 (6.2-8.4)	6.9 (6.0-8.1)	6.8 (5.9-8.0)	6.0 (5.1-7.1)
480-489	11.7 (10.1-15.6)	9.8 (8.5-11.2)	9.5 (8.2-11.6)	9.1 (7.9-10.4)	8.7 (7.7-10.3)	8.4 (7.1-9.6)	8.2 (7.1-9.8)	7.8 (6.8-9.0)	7.7 (6.6-9.0)	7.5 (6.6-8.7)	7.3 (6.5-8.4)	6.8 (5.9-8.1)	6.9 (5.8-8.1)	6.1 (5.2-7.2)
490-499	11.6 (10.7-13.2)	9.9 (8.7-11.5)	9.6 (8.3-11.5)	9.6 (8.2-11.4)	8.6 (7.7-10.2)	8.5 (7.4-9.8)	8.0 (7.2-9.1)	7.8 (6.8-8.9)	7.8 (6.7-9.1)	7.3 (6.5-8.6)	7.5 (6.3-8.6)	7.2 (6.2-8.1)	6.8 (5.9-8.2)	6.2 (5.2-7.2)
500-509	12.1 (10.3-14.8)	10.4 (8.7-12.3)	9.2 (7.9-10.9)	8.8 (8.0-10.2)	8.9 (7.6-10.2)	8.4 (7.4-10.0)	8.2 (7.2-9.7)	7.7 (6.8-8.9)	7.5 (6.5-8.9)	7.5 (6.5-8.5)	7.2 (6.3-8.4)	7.0 (6.1-7.9)	7.0 (5.8-7.9)	6.2 (5.2-7.2)
510-519	12.3 (10.7-14.0)	9.9 (8.7-11.9)	10.2 (8.9-11.7)	9.6 (8.4-11.1)	8.9 (7.6-10.7)	8.6 (7.6-10.1)	8.1 (6.9-9.6)	8.0 (7.1-9.3)	7.7 (6.8-9.1)	7.8 (6.8-8.8)	7.4 (6.6-8.4)	7.0 (6.0-8.2)	7.1 (5.9-8.1)	6.3 (5.4-7.3)
520-529	12.0 (10.3-15.9)	11.9 (9.0-14.2)	9.7 (8.3-11.6)	9.4 (8.5-11.3)	9.0 (8.0-10.5)	8.4 (7.3-10.0)	8.6 (7.6-10.2)	8.0 (7.0-9.3)	7.8 (6.8-8.9)	7.3 (6.4-8.5)	7.3 (6.5-8.7)	7.1 (6.3-8.3)	6.9 (6.0-8.1)	6.3 (5.3-7.3)
530-539	12.0 (9.8-14.8)	10.5 (9.4-12.0)	10.0 (9.0-11.7)	9.3 (8.3-10.2)	9.1 (7.9-10.9)	8.7 (7.5-10.4)	8.5 (7.4-9.8)	8.3 (7.3-9.7)	7.7 (6.8-9.1)	7.4 (6.4-8.8)	7.4 (6.2-8.7)	7.2 (6.2-8.6)	7.1 (6.0-8.1)	6.3 (5.3-7.3)
540-549	11.3 (9.8-14.4)	10.0 (9.3-12.8)	10.7 (9.0-13.2)	9.7 (8.3-11.2)	9.1 (7.8-10.0)	9.0 (7.9-10.2)	8.6 (7.6-10.0)	8.0 (6.9-9.4)	8.0 (6.7-9.3)	7.4 (6.4-8.6)	7.4 (6.5-9.0)	7.4 (6.4-8.3)	7.1 (6.2-8.3)	6.3 (5.4-7.4)
550-559	12.2 (10.4-15.9)	10.8 (10.2-12.1)	10.1 (9.1-11.1)	9.8 (8.5-12.0)	9.3 (8.2-10.8)	8.6 (7.6-10.5)	8.5 (7.5-10.0)	7.7 (6.9-9.1)	8.1 (6.8-9.1)	8.1 (6.9-9.2)	7.5 (6.6-8.6)	7.3 (6.3-8.4)	7.2 (6.2-8.1)	6.2 (5.2-7.3)
560-569	13.8 (11.3-17.2)	10.5 (9.0-11.8)	10.7 (9.0-13.2)	9.8 (8.7-12.3)	8.7 (7.8-10.6)	8.5 (7.5-10.5)	8.7 (7.5-9.9)	8.3 (7.2-9.5)	8.2 (7.1-9.5)	7.7 (6.8-8.8)	7.2 (6.3-8.3)	7.0 (6.3-7.9)	7.3 (6.0-8.3)	6.2 (5.3-7.3)
570-579	15.4 (11.6-19.9)	10.8 (10.4-14.6)	10.3 (9.1-12.3)	9.8 (8.3-12.0)	9.2 (8.0-11.3)	9.0 (7.8-10.8)	8.6 (7.3-9.9)	8.4 (7.3-10.0)	8.1 (7.0-9.2)	7.4 (6.4-8.7)	7.5 (6.6-8.4)	7.4 (6.5-8.4)	6.9 (6.1-7.9)	6.2 (5.2-7.3)
580-589	12.7 (11.6-17.7)	10.6 (9.6-13.3)	10.4 (9.2-11.5)	10.7 (8.2-11.5)	9.5 (8.6-10.9)	9.3 (7.9-11.1)	8.4 (7.5-10.0)	8.6 (7.7-10.1)	8.3 (7.1-9.2)	7.8 (6.8-8.9)	7.5 (6.4-8.7)	7.4 (6.3-8.6)	7.1 (5.9-8.0)	6.4 (5.4-7.6)
590-599	12.5 (10.8-19.3)	11.7 (10.3-12.6)	10.2 (9.0-11.6)	10.1 (9.0-11.2)	9.8 (8.6-12.2)	9.2 (8.1-11.0)	8.7 (7.8-11.0)	8.8 (7.5-10.4)	8.5 (7.6-9.9)	7.7 (6.6-9.0)	7.4 (6.5-8.8)	7.2 (6.6-8.9)	7.1 (6.0-8.2)	5.9 (5.1-7.4)
600-609	13.7 (11.8-22.3)	11.0 (10.0-13.2)	11.1 (9.2-14.8)	9.6 (8.7-11.4)	9.2 (8.2-10.9)	8.9 (7.7-10.3)	8.6 (7.6-9.7)	8.5 (7.2-10.0)	8.3 (7.2-9.1)	7.8 (6.8-9.0)	7.7 (6.7-8.8)	7.5 (6.5-8.3)	7.3 (6.2-8.2)	6.3 (5.3-7.3)
610-619	15.4 (11.6-18.1)	11.5 (10.4-14.3)	10.6 (9.1-13.4)	10.4 (8.5-13.0)	9.5 (8.2-11.5)	9.0 (8.3-10.2)	8.8 (7.4-10.1)	8.4 (7.3-10.1)	8.5 (7.3-10.0)	8.1 (6.9-9.4)	7.7 (6.4-9.1)	7.3 (6.2-8.4)	7.5 (6.4-8.6)	6.4 (5.4-7.5)
620-629	16.4 (12.2-22.5)	11.7 (11.2-15.3)	12.8 (10.7-14.5)	9.7 (8.8-11.9)	9.5 (8.3-11.1)	9.3 (8.4-9.9)	8.4 (7.5-10.8)	9.0 (8.1-10.3)	8.2 (7.0-9.7)	8.3 (7.1-9.7)	7.5 (6.8-9.1)	7.5 (6.3-9.0)	7.6 (6.4-8.8)	6.4 (5.3-7.6)
630-639	14.1 (12.2-16.4)	13.4 (10.3-16.6)	12.1 (10.3-14.7)	10.5 (8.6-12.2)	9.7 (8.9-11.5)	9.9 (7.9-12.1)	8.6 (7.5-10.7)	8.6 (7.7-9.5)	8.3 (7.2-9.9)	8.1 (7.2-9.3)	7.5 (6.6-8.9)	7.2 (6.2-8.8)	7.3 (6.2-8.5)	6.2 (5.4-7.2)

© 2021 Sajja A et al. *JAMA Network Open.*

640-649	14.8 (12.9-16.9)	11.5 (10.3-16.5)	11.9 (10.4-12.7)	9.5 (8.8-11.9)	10.1 (8.9-11.5)	8.9 (7.7-10.5)	8.7 (7.4-10.3)	8.3 (7.4-9.9)	7.7 (6.8-9.8)	8.2 (6.9-9.5)	7.7 (6.7-8.8)	7.5 (6.5-8.1)	7.1 (6.2-8.1)	6.6 (5.6-7.2)
650-659	14.2 (11.9-23.3)	12.2 (9.8-14.9)	11.0 (9.7-14.5)	10.6 (8.8-11.8)	9.8 (8.3-11.7)	9.1 (8.3-11.1)	9.0 (8.0-10.7)	9.1 (7.7-10.4)	8.2 (7.0-10.2)	8.0 (6.9-9.4)	7.4 (6.7-9.3)	7.6 (6.7-8.6)	7.5 (6.6-8.8)	6.4 (5.4-7.7)
660-669	15.0 (12.2-18.9)	14.6 (11.0-19.9)	11.3 (10.1-14.1)	9.8 (8.4-11.0)	9.5 (8.6-11.4)	9.2 (7.8-10.4)	8.9 (7.5-10.5)	8.2 (7.1-9.5)	8.2 (7.3-9.2)	8.4 (7.1-9.6)	7.5 (6.9-8.9)	7.5 (6.9-8.4)	7.5 (6.7-9.1)	6.5 (5.6-7.7)
670-679	14.2 (12.1-20.4)	12.3 (10.4-15.3)	10.6 (9.5-14.0)	10.3 (9.7-12.0)	9.4 (8.4-10.7)	9.5 (8.5-11.4)	8.7 (8.0-10.0)	8.8 (7.8-10.0)	8.3 (7.2-9.5)	8.5 (7.3-10.3)	7.8 (6.9-9.1)	7.8 (6.7-8.8)	7.4 (6.0-8.4)	6.7 (5.4-7.8)
680-689	16.7 (13.5-19.8)	11.6 (10.6-12.9)	11.3 (10.2-13.0)	11.6 (10.1-14.0)	10.0 (9.3-12.2)	9.8 (8.6-12.6)	9.5 (8.1-11.3)	8.8 (7.8-10.8)	8.0 (7.3-9.1)	7.8 (6.9-9.5)	7.6 (6.8-9.0)	7.4 (6.7-8.4)	7.2 (6.5-8.3)	6.7 (5.7-7.9)
690-699	15.0 (12.2-18.4)	12.9 (11.4-16.8)	12.6 (9.8-17.4)	10.8 (9.6-11.7)	10.2 (8.7-12.7)	9.5 (8.6-12.3)	9.9 (8.4-11.1)	8.7 (7.7-10.3)	8.3 (7.1-10.3)	8.1 (7.0-9.6)	8.1 (7.1-9.1)	7.6 (6.4-9.3)	7.7 (6.3-8.4)	6.5 (5.5-7.5)
700-709	16.6 (13.1-20.2)	13.1 (11.2-16.1)	11.0 (10.4-13.6)	10.7 (9.1-13.3)	9.7 (8.7-10.6)	9.4 (8.4-11.4)	9.5 (8.2-10.8)	9.1 (7.5-10.0)	7.9 (6.9-9.8)	8.4 (7.1-10.5)	8.5 (7.2-9.8)	7.6 (6.3-8.9)	7.7 (6.5-9.1)	6.9 (5.7-7.8)
710-719	14.5 (11.1-19.3)	11.7 (11.4-17.8)	10.7 (9.7-13.0)	10.7 (9.8-12.5)	9.9 (8.9-11.3)	9.1 (8.3-10.3)	9.8 (8.6-11.9)	8.6 (7.8-10.8)	8.7 (7.8-9.9)	8.1 (6.9-9.2)	8.0 (6.2-9.1)	7.6 (6.9-9.4)	7.6 (6.5-8.8)	6.4 (5.6-7.7)
720-729	16.5 (13.5-26.8)	12.9 (11.7-20.1)	11.0 (9.4-14.6)	11.8 (9.7-13.7)	9.5 (8.7-10.8)	9.7 (8.5-10.7)	9.3 (8.1-11.1)	8.9 (8.1-10.3)	8.3 (7.7-9.2)	8.4 (7.3-10.2)	7.8 (6.9-9.2)	7.2 (6.8-9.0)	7.8 (6.7-9.1)	6.6 (5.6-7.8)
730-739	18.2 (15.4-22.2)	13.2 (12.2-15.9)	12.2 (10.7-15.7)	11.6 (10.4-14.7)	10.0 (8.7-12.1)	10.5 (9.3-11.7)	9.4 (8.8-10.6)	9.3 (8.4-10.7)	8.5 (7.3-10.3)	8.5 (7.1-10.4)	8.5 (7.4-11.3)	7.9 (6.9-8.9)	7.5 (6.3-8.9)	6.6 (5.6-7.7)
740-749	17.5 (14.2-19.0)	12.1 (10.2-14.1)	11.7 (10.4-15.5)	11.9 (10.8-14.6)	11.2 (9.0-15.3)	9.9 (9.5-11.8)	9.2 (7.9-11.5)	9.2 (7.9-10.6)	8.5 (7.8-10.4)	7.9 (7.0-9.9)	8.1 (7.1-9.4)	8.1 (7.0-9.0)	7.5 (6.7-9.3)	6.6 (5.4-7.5)
750-759	17.5 (15.8-21.6)	13.6 (12.5-16.5)	13.5 (10.7-15.1)	12.1 (10.6-14.2)	12.6 (9.1-13.8)	9.8 (8.1-11.3)	10.1 (8.4-11.7)	9.1 (7.9-10.8)	9.0 (7.4-9.8)	8.4 (7.6-9.9)	8.2 (7.3-9.4)	8.7 (7.0-9.8)	7.4 (6.8-8.7)	6.4 (5.2-7.6)
760-769	19.2 (13.1-21.2)	13.7 (12.5-15.5)	12.5 (10.7-14.3)	10.9 (9.7-13.1)	10.3 (9.1-14.1)	9.7 (8.9-11.2)	10.5 (8.9-11.8)	9.6 (7.8-11.4)	8.7 (7.4-9.5)	8.1 (7.0-9.5)	8.7 (7.9-10.1)	7.4 (6.7-8.6)	8.2 (6.6-9.4)	6.5 (5.6-7.6)
770-779	17.3 (12.3-24.2)	14.1 (12.3-17.2)	13.4 (11.4-16.9)	12.9 (9.3-14.7)	10.5 (9.3-12.7)	10.2 (9.4-11.5)	10.0 (8.4-13.1)	8.7 (7.7-10.9)	8.2 (7.4-10.0)	9.3 (7.7-11.1)	8.2 (7.5-9.0)	8.0 (6.5-9.0)	8.3 (7.1-10.1)	6.7 (5.8-8.1)
780-789	23.9 (16.4-27.0)	14.1 (12.4-17.8)	12.4 (11.6-14.0)	11.5 (10.7-12.4)	10.4 (10.1-11.7)	10.4 (9.3-12.4)	10.4 (8.9-11.8)	9.3 (8.3-10.7)	9.0 (8.2-10.4)	8.2 (7.5-9.9)	8.8 (6.9-9.8)	8.1 (7.1-8.9)	7.4 (6.6-8.7)	6.7 (5.4-7.8)
790-799	15.6 (13.9-20.3)	18.5 (13.6-19.9)	13.9 (12.1-19.5)	12.2 (11.1-13.6)	11.3 (10.1-15.5)	10.7 (9.0-13.3)	9.6 (8.7-11.0)	9.4 (8.5-10.8)	8.7 (7.7-9.1)	8.1 (7.6-8.8)	8.0 (7.3-9.0)	8.1 (7.3-8.9)	7.5 (6.7-9.1)	6.7 (5.8-7.8)

© 2021 Sajja A et al. *JAMA Network Open.*

C. 1040-Cell

TG level, mg/dL	Non-HDL-C, mg/dL <100	100-104	105-109	110-114	115-119	120-124	125-129	130-134	135-139	140-144	145-149	150-154	155-159	160-164	165-169	170-174	175-179	180-184	185-189	190-194	195-199	200-204	205-209	210-214	215-219	220+
400-409	10.4 (9.1-12.5)	9.7 (8.3-11.1)	8.8 (7.5-10.5)	8.8 (7.7-10.4)	8.9 (7.7-10.2)	8.3 (7.3-9.8)	8.5 (7.4-9.6)	8.4 (7.4-9.5)	8.1 (7.1-9.4)	7.8 (6.7-9.1)	7.9 (6.8-9.0)	7.6 (6.5-8.8)	7.7 (6.8-8.7)	7.4 (6.5-8.6)	7.4 (6.4-8.5)	7.4 (6.6-8.5)	7.3 (6.3-8.3)	7.0 (6.2-8.1)	7.2 (6.2-8.2)	6.8 (5.9-7.6)	7.1 (6.2-7.9)	6.7 (5.9-7.7)	6.6 (5.8-7.6)	6.6 (5.6-7.7)	6.6 (5.9-7.7)	6.1 (5.1-6.9)
410-419	10.7 (9.4-12.7)	9.4 (8.6-11.1)	9.1 (8.0-10.2)	9.2 (8.1-11.1)	8.7 (7.5-10.2)	8.4 (7.6-9.9)	8.8 (7.4-9.8)	8.2 (7.3-10.0)	8.4 (7.4-9.5)	8.0 (7.1-9.1)	7.8 (6.8-9.0)	7.6 (6.6-8.7)	7.6 (6.7-9.0)	7.5 (6.5-8.6)	7.6 (6.6-8.8)	7.4 (6.4-8.4)	7.3 (6.4-8.3)	7.2 (6.3-8.2)	6.9 (6.1-8.0)	6.8 (6.0-7.8)	7.0 (6.2-8.0)	6.9 (5.8-8.0)	6.6 (5.9-7.7)	6.6 (5.8-7.6)	6.3 (5.7-7.4)	6.0 (5.1-7.0)
420-429	10.3 (9.2-13.7)	9.3 (7.7-10.8)	8.8 (7.8-10.4)	9.6 (8.1-11.7)	8.9 (8.2-10.0)	8.9 (7.7-10.1)	8.6 (7.5-10.0)	8.6 (7.4-9.7)	8.3 (6.9-9.6)	7.8 (6.9-9.1)	7.9 (7.0-8.9)	7.6 (6.8-9.0)	7.8 (6.5-9.1)	7.8 (6.8-8.5)	7.5 (6.6-8.5)	7.4 (6.4-8.5)	7.3 (6.2-8.5)	7.1 (6.2-8.2)	7.0 (6.1-8.1)	7.1 (6.2-8.0)	6.9 (5.9-7.6)	6.9 (6.1-8.0)	6.7 (5.9-7.7)	6.9 (5.8-7.8)	6.3 (5.6-7.1)	6.0 (5.1-7.0)
430-439	11.2 (9.2-14.0)	9.2 (7.8-11.8)	9.0 (8.0-11.2)	9.9 (8.6-11.4)	8.6 (7.4-10.3)	8.9 (7.8-10.3)	8.6 (7.2-9.9)	8.3 (7.3-9.8)	8.4 (7.4-9.5)	8.1 (7.0-9.6)	8.0 (6.9-9.3)	7.9 (6.8-9.3)	7.8 (6.5-8.9)	7.8 (6.5-8.9)	7.4 (6.5-8.7)	7.4 (6.5-8.2)	7.2 (6.2-8.5)	7.2 (6.5-8.2)	7.1 (6.2-8.3)	7.2 (6.3-8.2)	6.9 (6.1-7.7)	6.9 (5.9-7.7)	6.7 (5.6-7.5)	6.6 (5.6-7.6)	6.5 (5.6-7.6)	6.0 (5.1-6.9)
440-449	12.0 (10.3-13.9)	9.2 (8.2-10.9)	9.2 (8.5-10.8)	9.5 (8.3-10.6)	9.1 (7.9-10.4)	8.9 (7.8-10.4)	8.4 (7.3-9.7)	8.2 (7.0-9.4)	8.1 (7.3-9.4)	8.3 (7.4-9.5)	8.3 (7.2-9.4)	7.9 (7.0-9.1)	7.8 (6.7-9.0)	7.8 (6.7-9.0)	7.3 (6.2-8.7)	7.6 (6.5-8.6)	7.3 (6.3-8.3)	7.4 (6.3-8.4)	7.6 (6.3-8.8)	7.0 (5.9-8.2)	7.0 (6.0-8.0)	6.8 (5.9-7.8)	7.0 (6.1-7.8)	6.7 (5.6-7.5)	6.6 (5.6-7.4)	6.0 (5.1-7.1)
450-459	11.3 (9.6-13.4)	10.1 (8.4-12.6)	9.7 (8.6-11.0)	9.8 (8.9-10.8)	8.9 (7.6-10.5)	8.8 (7.9-10.7)	9.4 (8.0-10.5)	8.4 (7.3-10.1)	8.4 (7.3-9.4)	8.3 (7.3-9.4)	7.8 (7.0-9.0)	8.1 (7.1-9.0)	8.0 (6.8-9.2)	7.7 (6.6-8.6)	7.8 (6.8-9.2)	7.2 (6.3-8.4)	7.3 (6.3-8.5)	7.2 (6.2-8.5)	7.2 (6.1-8.4)	7.3 (6.3-8.3)	7.1 (6.3-8.3)	7.1 (6.2-8.1)	6.9 (6.1-7.8)	6.8 (6.0-7.9)	6.9 (5.9-7.6)	6.0 (5.2-7.0)
460-469	12.3 (10.2-14.2)	10.4 (9.0-12.5)	9.6 (8.3-10.7)	9.1 (7.9-10.7)	9.5 (8.1-11.0)	9.0 (7.9-11.0)	8.7 (7.8-10.6)	9.0 (7.6-10.4)	8.1 (7.2-9.7)	8.5 (7.3-10.0)	8.4 (7.2-9.5)	8.0 (7.2-9.1)	7.9 (7.1-9.2)	7.8 (6.9-8.9)	7.7 (6.4-9.0)	7.8 (6.8-8.9)	7.8 (6.5-8.8)	7.5 (6.4-8.6)	7.5 (6.5-8.5)	7.2 (6.1-8.2)	6.9 (6.0-8.1)	6.9 (5.8-8.1)	7.0 (6.0-7.9)	6.6 (5.7-7.5)	6.8 (5.5-7.7)	6.1 (5.1-7.1)
470-479	10.6 (9.3-13.1)	10.0 (9.1-11.4)	10.1 (9.0-11.9)	9.6 (8.1-10.6)	8.8 (7.8-10.3)	8.7 (8.0-10.2)	9.2 (8.2-10.6)	8.8 (7.2-10.1)	8.5 (7.6-9.7)	8.5 (7.4-10.3)	8.1 (7.1-9.6)	7.7 (7.1-9.4)	8.4 (7.3-9.7)	7.9 (7.0-9.0)	7.7 (6.7-9.3)	7.9 (7.0-9.1)	7.5 (6.6-8.8)	7.2 (6.2-8.4)	7.2 (6.2-8.5)	7.3 (6.2-8.5)	7.2 (6.1-8.1)	7.1 (6.1-8.1)	6.8 (6.0-8.1)	6.8 (5.9-7.8)	6.8 (6.1-8.0)	6.0 (5.1-7.1)
480-489	11.7 (10.1-15.6)	10.1 (9.0-11.2)	9.6 (8.3-12.0)	9.2 (8.3-11.1)	10.1 (8.0-11.8)	9.3 (8.3-10.8)	8.9 (7.7-10.1)	8.8 (8.0-10.7)	8.6 (7.5-10.0)	8.6 (7.3-9.7)	7.9 (6.9-9.3)	8.4 (7.3-10.0)	8.2 (7.1-9.5)	7.8 (6.8-8.9)	7.7 (6.9-9.4)	7.5 (6.6-9.0)	7.7 (6.6-9.1)	7.6 (6.6-8.9)	7.3 (6.6-8.6)	7.3 (6.7-8.4)	7.3 (6.3-8.4)	6.8 (6.0-8.1)	6.7 (5.8-8.1)	7.0 (6.2-8.1)	6.7 (5.5-8.1)	6.1 (5.2-7.2)
490-499	11.6 (10.7-13.2)	9.4 (8.7-11.5)	10.0 (8.8-11.2)	10.7 (9.3-12.6)	9.2 (7.9-11.0)	9.6 (8.4-11.5)	9.6 (8.0-11.3)	9.1 (7.9-10.4)	8.5 (7.5-9.5)	8.6 (7.6-10.2)	8.2 (7.4-9.7)	7.8 (7.2-9.0)	8.1 (7.2-9.1)	7.9 (6.9-9.5)	7.7 (6.7-8.6)	7.9 (6.6-9.2)	7.7 (6.8-9.0)	7.3 (6.3-8.6)	7.4 (6.6-8.6)	7.5 (6.1-8.8)	7.5 (6.3-8.6)	7.2 (6.2-7.9)	7.1 (6.4-8.2)	6.9 (6.0-8.2)	6.8 (5.6-8.2)	6.2 (5.2-7.2)
500-509	12.1 (10.3-14.8)	10.4 (8.7-11.5)	10.1 (8.7-12.3)	8.9 (7.8-9.9)	9.4 (8.5-11.1)	8.8 (8.1-10.2)	8.8 (7.8-10.3)	9.1 (7.8-9.8)	8.8 (7.6-10.5)	8.6 (7.4-10.1)	8.1 (7.4-9.7)	8.1 (7.3-9.5)	8.3 (7.2-9.8)	8.1 (7.1-9.1)	7.4 (6.7-8.6)	7.5 (6.5-9.2)	7.4 (6.5-8.6)	7.5 (6.7-8.7)	7.4 (6.3-8.4)	7.2 (6.2-8.2)	7.3 (6.3-8.5)	7.1 (6.2-7.9)	7.0 (6.0-8.0)	7.2 (6.1-8.5)	6.6 (5.5-7.8)	6.2 (5.2-7.2)
510-519	12.3 (10.7-14.0)	10.0 (8.7-13.5)	9.7 (8.8-11.2)	10.3 (9.3-11.7)	9.9 (8.6-11.7)	10.2 (8.8-12.6)	9.1 (8.1-10.5)	8.9 (7.9-10.3)	8.9 (7.5-11.0)	8.7 (7.6-10.2)	8.3 (7.5-9.8)	8.5 (7.2-9.8)	7.8 (6.6-9.2)	7.9 (7.0-9.1)	8.2 (7.1-9.4)	8.0 (6.7-9.6)	7.6 (6.9-8.8)	7.9 (7.0-9.1)	7.6 (6.5-8.7)	7.6 (6.8-8.7)	7.1 (6.3-8.1)	7.1 (6.0-8.1)	6.7 (5.8-8.2)	7.2 (6.1-8.1)	7.0 (5.7-8.0)	6.3 (5.4-7.3)
520-529	12.0 (10.3-15.9)	10.7 (8.8-14.2)	12.2 (9.2-14.2)	9.7 (8.3-11.6)	9.7 (8.4-11.3)	9.6 (8.5-11.1)	9.4 (8.7-11.7)	8.7 (7.9-10.1)	9.4 (8.3-10.9)	8.9 (7.4-11.0)	8.1 (7.2-9.2)	8.5 (7.6-10.3)	8.7 (7.5-10.2)	7.9 (7.0-9.4)	8.1 (7.0-9.3)	7.9 (6.9-8.9)	7.8 (6.7-8.8)	7.7 (6.9-8.9)	7.1 (6.1-8.1)	7.3 (6.6-8.7)	7.3 (6.4-8.8)	7.3 (6.4-8.3)	7.1 (6.2-8.2)	6.8 (6.0-7.8)	7.6 (6.1-8.4)	6.3 (5.3-7.3)
530-539	12.0 (9.8-14.8)	10.9 (9.1-12.6)	10.2 (9.4-11.5)	10.2 (9.1-11.7)	9.7 (8.8-11.6)	9.2 (8.3-10.0)	9.4 (8.3-11.5)	8.6 (7.6-9.9)	9.7 (8.3-11.1)	9.1 (7.9-11.0)	8.6 (7.1-10.4)	8.4 (7.4-9.5)	8.6 (7.3-10.1)	8.3 (7.3-9.8)	8.2 (7.1-9.6)	7.7 (6.9-9.4)	7.7 (6.7-8.7)	7.5 (6.6-9.5)	6.9 (6.3-8.4)	7.6 (6.4-8.8)	7.0 (6.2-8.6)	7.2 (6.2-8.3)	7.4 (6.2-8.9)	7.0 (6.2-8.1)	7.1 (5.4-8.1)	6.3 (5.3-7.3)
540-549	11.3 (9.8-14.4)	10.7 (9.5-13.0)	9.6 (8.8-11.6)	10.1 (9.1-11.4)	11.2 (9.0-14.1)	9.9 (8.0-11.8)	9.6 (8.6-10.7)	8.8 (7.7-10.0)	9.2 (7.8-9.9)	9.1 (7.9-10.3)	8.9 (7.8-10.2)	8.7 (7.6-9.9)	8.4 (7.5-10.0)	8.3 (6.9-9.5)	7.7 (7.0-9.4)	8.3 (7.1-9.4)	7.7 (6.5-9.1)	7.4 (6.5-9.0)	7.2 (6.4-8.1)	7.8 (6.4-9.2)	7.4 (6.6-8.8)	7.6 (6.4-8.2)	6.9 (6.3-9.0)	7.1 (6.1-8.3)	7.0 (6.3-8.3)	6.3 (5.4-7.4)
550-559	12.2 (10.4-15.9)	10.8 (10.6-11.8)	10.6 (9.9-12.1)	10.1 (9.1-11.0)	10.0 (9.0-11.2)	9.9 (9.0-12.0)	9.3 (8.4-11.9)	9.4 (8.4-10.9)	9.2 (8.1-10.8)	8.8 (7.6-10.8)	8.4 (7.6-10.4)	8.3 (7.5-9.2)	8.9 (7.6-10.5)	7.7 (7.1-9.2)	7.7 (6.8-8.9)	8.4 (7.1-9.7)	7.7 (6.6-8.6)	8.3 (6.9-9.3)	8.0 (6.9-9.2)	7.4 (6.7-8.7)	7.5 (6.5-8.4)	7.4 (6.7-8.4)	7.2 (6.0-8.6)	7.4 (6.6-8.3)	6.9 (5.9-7.8)	6.2 (5.2-7.3)

© 2021 Sajja A et al. *JAMA Network Open.*

560-569	13.8 (11.3-17.2)	10.6 (8.9-11.7)	9.8 (9.0-11.9)	10.5 (9.0-13.8)	11.1 (10.2-13.0)	9.9 (9.1-12.3)	9.5 (8.3-10.9)	8.8 (7.9-11.0)	8.6 (7.6-9.8)	9.6 (7.8-10.5)	8.2 (7.4-10.1)	8.6 (7.6-10.1)	9.0 (7.3-9.8)	8.2 (7.2-9.6)	8.4 (7.3-9.4)	8.5 (7.2-10.0)	7.8 (6.9-9.0)	7.8 (6.9-8.8)	7.6 (6.8-8.5)	7.2 (6.2-8.2)	7.3 (6.3-8.5)	7.0 (6.0-8.0)	7.1 (6.6-7.9)	7.3 (6.0-8.4)	7.2 (6.1-8.3)	6.2 (5.3-7.3)
570-579	15.4 (11.6-19.9)	11.1 (10.6-17.9)	10.6 (10.2-11.8)	11.1 (9.7-13.0)	10.2 (8.9-11.3)	10.3 (8.3-12.9)	9.4 (8.3-10.8)	10.9 (8.7-12.3)	8.8 (7.8-9.9)	9.3 (7.9-11.3)	8.8 (7.8-10.6)	8.8 (7.4-10.1)	8.2 (7.2-9.4)	8.0 (7.1-10.2)	8.6 (7.5-9.9)	8.2 (7.2-9.5)	8.1 (6.7-9.0)	7.2 (6.4-9.0)	7.5 (6.4-8.5)	7.7 (6.5-8.6)	7.4 (6.6-8.4)	7.5 (6.6-8.2)	7.4 (6.2-8.6)	6.9 (6.1-7.6)	7.1 (6.0-8.1)	6.2 (5.2-7.3)
580-589	12.7 (11.6-17.7)	11.9 (10.1-14.3)	10.4 (8.9-12.5)	10.4 (9.7-12.0)	10.4 (9.2-11.2)	10.9 (8.8-11.4)	9.8 (8.0-11.7)	9.4 (8.8-11.0)	9.7 (7.9-10.6)	9.5 (7.9-11.1)	9.0 (7.9-11.1)	8.7 (7.5-10.2)	8.2 (7.5-10.0)	8.6 (7.8-10.2)	8.5 (7.5-10.1)	8.3 (6.9-9.1)	8.3 (7.4-9.3)	7.4 (6.5-8.8)	8.2 (7.3-9.1)	7.5 (6.4-8.7)	7.3 (6.4-8.5)	7.5 (6.3-8.8)	7.1 (6.1-8.2)	7.3 (5.7-8.4)	7.0 (6.1-7.8)	6.4 (5.4-7.6)
590-599	12.5 (10.8-19.3)	11.7 (10.1-12.6)	11.1 (10.3-12.9)	10.0 (8.7-14.1)	10.4 (9.2-11.0)	9.6 (8.7-10.6)	10.6 (9.5-11.5)	9.9 (9.0-11.6)	9.4 (7.8-12.2)	8.6 (7.9-10.1)	9.4 (8.1-11.1)	8.7 (7.9-11.2)	8.6 (7.8-10.8)	8.4 (7.4-10.3)	8.8 (7.5-10.6)	8.5 (7.6-9.7)	8.3 (7.5-10.2)	7.7 (6.9-8.9)	7.5 (6.3-9.6)	7.5 (6.5-8.8)	7.2 (6.5-8.8)	7.3 (6.6-8.6)	7.0 (6.5-9.0)	7.7 (6.6-9.2)	6.7 (5.9-7.9)	5.9 (5.1-7.4)
600-609	13.7 (11.8-22.3)	11.5 (10.0-12.9)	10.9 (9.9-13.2)	10.9 (9.1-14.1)	11.8 (9.8-14.8)	9.6 (8.4-11.1)	9.6 (8.8-11.6)	9.1 (8.2-10.7)	9.4 (8.0-11.8)	9.5 (8.2-11.0)	8.5 (7.7-9.8)	8.1 (7.4-9.0)	9.0 (7.9-9.9)	8.5 (7.3-10.0)	8.3 (6.9-10.1)	8.6 (7.6-9.1)	8.0 (7.1-9.0)	8.2 (7.1-9.2)	7.6 (6.6-8.7)	7.5 (6.7-8.6)	8.0 (6.5-9.4)	7.8 (7.0-9.0)	7.3 (6.3-8.1)	7.0 (6.1-8.2)	7.5 (6.3-8.2)	6.3 (5.3-7.3)
610-619	15.4 (11.6-18.1)	11.7 (10.4-13.9)	11.4 (9.9-15.0)	12.1 (10.3-17.4)	9.6 (9.0-12.5)	10.0 (9.2-12.0)	10.4 (8.4-13.7)	9.7 (8.6-10.9)	9.5 (7.9-11.8)	9.3 (8.3-10.2)	8.9 (8.2-10.0)	8.8 (7.3-9.9)	8.8 (7.4-10.4)	8.6 (7.4-10.1)	8.2 (7.1-10.1)	8.4 (7.4-10.0)	8.6 (7.1-9.7)	8.3 (7.1-9.8)	7.6 (6.7-9.3)	7.5 (6.2-8.9)	8.0 (6.8-9.5)	7.3 (6.1-8.6)	7.3 (6.5-8.2)	7.6 (6.5-8.8)	7.4 (6.4-8.3)	6.4 (5.4-7.5)
620-629	16.4 (12.2-22.5)	11.8 (11.4-12.3)	11.6 (11.2-15.3)	13.2 (9.4-15.3)	12.7 (11.0-14.5)	9.9 (8.8-12.8)	9.5 (8.9-11.7)	9.6 (8.4-10.9)	9.3 (8.3-11.1)	9.2 (8.4-9.8)	9.3 (8.6-10.2)	8.4 (7.8-11.5)	8.4 (7.4-10.1)	9.2 (8.3-10.2)	8.7 (7.5-10.3)	8.5 (7.7-10.3)	7.8 (6.8-9.3)	8.1 (7.1-9.6)	8.6 (7.2-10.7)	7.5 (6.8-8.5)	7.4 (6.8-9.5)	7.7 (6.6-9.2)	6.8 (5.9-8.7)	7.7 (6.4-9.6)	7.4 (6.2-8.0)	6.4 (5.3-7.6)
630-639	14.1 (12.2-16.4)	12.4 (10.2-14.4)	14.2 (10.8-21.2)	12.6 (10.6-15.9)	11.6 (9.8-12.9)	12.4 (11.5-14.2)	9.8 (8.2-10.8)	9.6 (8.4-10.6)	9.9 (9.1-12.4)	9.6 (8.2-10.8)	9.9 (7.9-12.4)	8.8 (7.6-10.7)	8.6 (7.5-10.6)	8.6 (7.8-9.8)	8.2 (7.5-9.3)	8.2 (6.9-10.0)	8.5 (7.2-9.9)	8.0 (7.3-9.4)	8.1 (7.1-9.3)	7.6 (6.9-9.2)	7.4 (6.4-8.6)	7.2 (6.4-8.8)	7.2 (6.2-8.1)	6.8 (6.1-8.2)	7.7 (6.7-8.5)	6.2 (5.4-7.2)
640-649	14.8 (12.9-16.9)	13.8 (11.4-17.0)	10.6 (10.1-15.0)	11.9 (10.6-14.2)	11.6 (10.0-12.4)	10.3 (7.9-12.6)	9.3 (8.9-11.8)	10.0 (8.5-11.3)	10.2 (9.0-11.7)	8.9 (7.9-10.7)	8.9 (7.5-10.4)	8.9 (7.7-10.3)	8.0 (7.1-10.0)	8.2 (7.4-8.8)	8.6 (7.3-10.9)	7.8 (6.9-9.9)	7.6 (6.7-9.0)	8.0 (6.7-8.9)	8.5 (7.3-10.0)	7.6 (7.0-8.7)	7.8 (6.4-9.2)	7.5 (6.6-7.9)	7.5 (6.5-8.4)	6.8 (5.9-7.8)	7.6 (6.4-8.4)	6.6 (5.6-7.2)
650-659	14.2 (11.9-23.3)	11.3 (9.5-14.9)	12.4 (10.2-15.0)	10.3 (9.1-14.3)	11.9 (10.4-14.5)	9.3 (8.8-11.1)	10.8 (10.1-12.2)	11.7 (9.2-13.3)	8.7 (8.2-10.5)	9.4 (8.0-11.2)	9.1 (8.5-10.9)	8.7 (8.0-10.8)	9.3 (8.0-10.7)	9.2 (8.0-10.5)	9.0 (7.6-10.2)	8.5 (7.5-10.2)	7.6 (6.7-10.1)	8.3 (6.9-9.9)	7.8 (7.0-8.7)	7.3 (6.5-9.7)	7.5 (7.0-8.3)	7.4 (6.1-8.4)	8.0 (6.7-8.7)	7.7 (6.9-9.3)	6.9 (6.4-8.2)	6.4 (5.4-7.7)
660-669	15.0 (12.2-18.9)	18.5 (14.3-22.2)	13.1 (9.6-17.0)	10.7 (10.2-12.7)	11.9 (10.0-14.1)	11.1 (8.9-11.3)	9.8 (8.4-10.2)	9.4 (8.6-12.5)	9.5 (8.6-11.2)	9.7 (8.0-13.1)	8.8 (7.6-9.2)	9.4 (7.6-10.5)	8.8 (7.4-11.0)	8.0 (6.9-9.5)	8.4 (7.1-9.9)	8.3 (7.5-9.7)	8.1 (7.2-8.8)	8.6 (7.0-9.9)	8.3 (7.5-9.2)	7.1 (6.8-8.9)	7.7 (7.0-8.8)	7.9 (7.4-8.9)	7.1 (6.3-8.0)	7.5 (6.9-9.1)	7.2 (6.5-8.5)	6.5 (5.6-7.7)
670-679	14.2 (12.1-20.4)	11.2 (10.2-13.0)	12.8 (11.0-16.2)	10.6 (9.9-12.4)	12.2 (9.1-16.7)	9.7 (8.8-11.4)	11.4 (10.0-14.1)	9.4 (7.8-9.9)	9.8 (8.5-12.7)	10.3 (8.6-12.7)	9.1 (8.4-10.5)	8.6 (8.3-11.2)	8.9 (7.8-10.0)	8.8 (8.1-10.4)	9.0 (7.7-9.7)	8.3 (7.3-9.5)	8.3 (7.1-9.4)	8.5 (7.3-10.5)	8.5 (7.5-10.3)	7.4 (6.8-9.0)	8.3 (7.1-9.4)	7.8 (6.9-8.7)	6.8 (6.3-9.3)	7.1 (6.0-8.2)	7.5 (5.9-8.4)	6.7 (5.4-7.8)
680-689	16.7 (13.5-19.8)	10.9 (10.6-11.1)	12.4 (12.2-12.9)	11.6 (10.2-13.9)	10.8 (10.3-11.4)	11.4 (10.9-14.0)	12.4 (9.8-14.2)	9.5 (9.1-12.2)	10.4 (9.5-11.6)	9.1 (8.5-12.2)	9.8 (8.7-13.1)	9.0 (8.1-11.2)	9.7 (8.1-12.3)	8.3 (7.2-9.9)	9.1 (8.2-11.1)	7.7 (6.9-9.7)	8.0 (7.3-8.7)	7.5 (6.7-10.5)	7.9 (6.9-9.4)	7.9 (6.6-8.8)	7.5 (6.8-9.1)	7.2 (6.6-8.0)	7.9 (6.9-8.5)	7.3 (6.5-8.6)	7.2 (6.5-8.2)	6.7 (5.7-7.9)
690-699	15.0 (12.2-18.4)	16.6 (11.7-19.3)	11.1 (10.8-11.8)	14.8 (12.2-20.3)	11.3 (9.3-13.8)	10.3 (9.6-11.9)	10.9 (9.4-11.5)	11.3 (9.3-13.7)	9.0 (7.9-10.8)	9.7 (8.6-12.4)	9.4 (8.5-12.2)	10.2 (8.4-11.1)	9.4 (7.9-10.8)	8.8 (7.3-9.1)	8.7 (7.8-10.3)	8.6 (6.9-11.0)	8.0 (7.1-9.2)	7.9 (7.2-9.2)	8.2 (6.6-10.3)	7.5 (6.8-9.6)	8.2 (7.5-9.0)	7.6 (6.0-9.1)	7.6 (6.7-9.3)	7.8 (6.6-8.3)	7.4 (6.2-9.8)	6.5 (5.5-7.5)
700-709	16.6 (13.1-20.2)	13.1 (11.2-35.3)	13.0 (11.4-15.7)	11.1 (10.1-17.5)	11.0 (10.6-13.4)	11.4 (9.0-13.0)	10.2 (9.1-13.6)	9.5 (8.7-10.3)	10.2 (8.8-11.9)	9.4 (8.2-11.3)	9.7 (8.6-11.4)	9.5 (8.2-11.2)	9.5 (8.3-10.4)	9.2 (7.9-10.4)	8.4 (6.9-9.2)	7.9 (7.3-9.5)	7.7 (6.7-10.6)	8.1 (7.2-10.3)	8.6 (6.9-10.5)	8.8 (7.3-10.2)	8.1 (6.5-9.3)	7.4 (6.0-8.5)	7.6 (7.1-8.9)	7.4 (6.5-9.1)	7.9 (6.5-9.1)	6.9 (5.7-7.8)
710-719	14.5 (11.1-19.3)	11.3 (11.3-11.3)	11.8 (11.5-23.8)	11.9 (10.3-16.0)	9.8 (8.8-10.9)	10.6 (9.9-14.0)	11.5 (9.8-12.1)	10.4 (9.0-11.9)	9.9 (8.6-10.7)	8.7 (8.2-9.4)	10.2 (9.0-10.9)	9.7 (8.5-11.9)	9.9 (8.9-11.5)	8.8 (7.4-10.8)	8.6 (8.2-10.1)	8.6 (7.8-9.6)	9.0 (7.8-10.0)	8.1 (7.2-9.3)	8.0 (6.8-9.1)	8.2 (7.5-9.0)	7.8 (6.1-9.3)	7.3 (6.2-8.5)	8.2 (7.0-10.0)	7.8 (6.6-8.5)	7.3 (6.5-9.9)	6.4 (5.6-7.7)
720-729	16.5 (13.5-26.8)	15.9 (11.7-20.1)	12.9 (10.4-21.2)	14.6 (13.7-17.2)	10.1 (9.3-11.3)	10.1 (9.7-12.9)	11.9 (11.1-13.7)	9.5 (8.8-10.5)	9.4 (8.1-11.0)	10.6 (8.8-11.4)	9.5 (8.3-10.1)	10.5 (8.7-12.6)	8.7 (7.8-10.4)	8.9 (8.3-10.3)	8.6 (7.4-10.3)	8.1 (7.4-8.8)	8.4 (8.1-10.3)	8.9 (7.7-10.8)	8.1 (7.2-9.5)	7.8 (7.0-9.1)	8.0 (6.8-9.7)	7.4 (7.1-8.8)	7.0 (6.6-9.0)	8.1 (6.9-8.9)	7.6 (6.7-9.2)	6.6 (5.6-7.8)
730-739	18.2 (15.4-22.2)	12.8 (12.4-13.1)	13.9 (12.0-17.1)	12.2 (10.7-15.7)	11.8 (10.7-16.3)	11.0 (9.8-14.1)	12.2 (11.2-16.7)	9.8 (8.4-11.9)	11.5 (9.2-12.2)	11.0 (9.8-13.3)	9.8 (9.2-11.5)	9.9 (8.9-11.3)	9.2 (8.4-9.9)	9.4 (8.8-11.2)	9.1 (7.8-10.3)	8.0 (6.8-11.4)	8.5 (7.8-9.5)	8.9 (7.4-10.9)	8.3 (7.1-10.2)	9.4 (8.1-11.4)	8.3 (7.4-8.7)	7.5 (7.0-8.9)	7.9 (6.8-8.9)	7.1 (6.0-9.1)	8.1 (6.7-8.8)	6.6 (5.6-7.7)

© 2021 Sajja A et al. *JAMA Network Open.*

740-749	17.5 (14.2-19.0)	12.1 (9.9-14.4)	12.1 (10.5-13.8)	11.8 (10.5-15.5)	10.7 (9.4-15.8)	12.3 (9.9-14.6)	11.9 (10.8-12.0)	11.8 (9.6-15.8)	9.4 (9.0-11.2)	9.8 (9.5-12.6)	9.9 (8.3-10.9)	9.8 (8.0-11.2)	8.5 (7.9-11.5)	9.2 (7.7-11.0)	9.1 (8.0-10.1)	8.6 (7.7-13.0)	8.4 (7.9-9.6)	7.6 (6.9-9.9)	8.1 (7.0-9.8)	8.0 (6.6-8.8)	8.1 (7.4-10.2)	8.3 (7.3-9.1)	8.1 (7.0-8.8)	7.6 (6.7-9.6)	7.4 (6.4-9.3)	6.6 (5.4-7.5)
750-759	17.5 (15.8-21.6)	18.9 (13.6-24.3)	13.0 (12.5-15.7)	15.0 (10.4-15.1)	13.0 (10.7-18.0)	11.6 (9.4-16.1)	12.6 (10.9-14.0)	12.6 (9.1-13.5)	13.0 (8.7-15.7)	9.8 (9.3-12.6)	9.5 (7.6-10.5)	9.5 (8.4-11.0)	10.4 (8.5-12.7)	8.7 (7.7-11.3)	9.4 (8.2-10.5)	8.5 (8.0-9.8)	9.0 (7.3-10.2)	8.4 (7.4-9.9)	9.0 (8.0-9.9)	7.9 (7.3-8.6)	8.3 (7.5-9.8)	8.9 (7.1-9.8)	7.7 (6.8-10.0)	7.3 (6.8-8.2)	7.6 (5.9-9.5)	6.4 (5.2-7.6)
760-769	19.2 (13.1-21.2)	13.7 (13.7-13.7)	14.0 (12.5-15.5)	12.3 (10.8-14.3)	12.5 (10.7-14.4)	11.2 (9.7-14.8)	10.9 (9.6-13.1)	11.9 (9.1-14.7)	10.3 (9.1-13.8)	9.9 (8.6-10.3)	9.6 (9.0-11.2)	11.6 (11.2-12.4)	9.3 (8.3-10.1)	9.3 (7.8-12.0)	9.9 (7.8-10.9)	7.8 (6.9-8.7)	9.3 (8.4-11.1)	8.3 (7.0-9.3)	7.7 (7.0-9.5)	8.4 (8.0-9.7)	9.1 (7.8-10.4)	7.4 (6.9-8.6)	7.3 (6.6-8.9)	8.2 (6.0-9.3)	8.4 (7.4-9.4)	6.5 (5.6-7.6)
770-779	17.3 (12.3-24.2)	14.4 (12.3-17.2)	13.4 (11.9-18.1)	13.2 (12.3-16.9)	13.4 (10.2-15.2)	10.4 (10.2-13.6)	13.2 (9.3-14.7)	12.3 (9.9-13.1)	9.9 (9.1-11.8)	10.5 (9.3-11.9)	9.9 (9.6-11.4)	10.4 (8.9-13.5)	9.4 (8.2-11.6)	9.8 (7.9-11.0)	8.3 (7.4-9.8)	8.5 (7.3-10.5)	8.2 (7.6-9.1)	8.7 (7.8-11.0)	9.9 (7.3-11.6)	8.2 (7.8-9.0)	8.1 (6.9-9.0)	8.2 (6.8-10.1)	6.9 (6.1-8.7)	8.5 (7.9-10.1)	7.6 (6.7-11.1)	6.7 (5.8-8.1)
780-789	23.9 (16.4-27.0)	14.1 (12.8-15.4)	15.1 (11.2-18.0)	12.4 (11.6-14.0)	12.7 (10.6-15.8)	12.1 (11.9-12.5)	10.9 (10.6-12.4)	10.1 (9.8-10.4)	11.3 (10.3-11.9)	11.2 (9.2-12.9)	10.1 (9.3-10.8)	10.0 (8.8-11.6)	10.6 (9.4-12.0)	9.2 (8.4-11.8)	9.3 (7.8-10.0)	9.1 (8.4-10.5)	9.0 (8.2-10.2)	8.3 (7.5-9.8)	8.1 (7.2-10.0)	9.1 (7.7-9.8)	7.7 (6.9-8.8)	8.0 (7.1-8.9)	8.4 (7.1-8.9)	7.4 (6.3-8.2)	7.4 (7.2-10.1)	6.7 (5.4-7.8)
790-799	15.6 (13.9-20.3)	13.6 (12.2-27.5)	19.2 (18.5-19.9)	14.1 (13.0-16.8)	13.0 (10.8-20.3)	12.3 (12.1-16.4)	11.7 (9.8-13.6)	13.7 (9.6-17.3)	11.2 (10.2-13.4)	10.9 (9.0-13.5)	9.8 (9.1-10.7)	9.8 (9.0-11.0)	9.6 (8.5-12.5)	9.5 (8.9-10.9)	9.3 (8.3-10.7)	9.0 (7.8-10.7)	8.6 (7.1-8.8)	8.1 (7.9-8.9)	8.3 (7.5-8.4)	7.9 (7.0-8.5)	8.5 (7.7-9.2)	7.7 (7.2-8.2)	8.5 (8.1-9.1)	7.4 (6.7-8.4)	9.0 (6.9-9.6)	6.7 (5.8-7.8)

© 2021 Sajja A et al. *JAMA Network Open.*

eTable 2. Accuracy in LDL-C Classification by Martin/Hopkins Method by 240 vs 560 vs 1040-Cell Counts

LDL-C, mg/dL	Martin/Hopkins LDL-C Estimation (%)	Extended Martin/Hopkins LDL-C (40 TG, 6 Non-HDL categories; 240 cells) Estimation (%)	Extended Martin/Hopkins LDL-C (40 TG, 14 Non-HDL categories; 560 cells) Estimation (%)	Extended Martin/Hopkins LDL-C (40 TG, 26 Non-HDL categories; 1040 cells) Estimation (%)
<40	40.1	57.2	59.9	60.2
40-69	66.4	67.8	69.0	68.2
70-99	65.7	65.2	65.5	65.0
100-129	61.0	60.7	60.8	60.6
130-159	57.2	58.5	58.4	58.1
160-189	53.3	56.0	56.0	56.0
≥190	65.3	70.1	70.1	70.1
Overall	61.0	62.1	62.3	62.0

© 2021 Sajja A et al. *JAMA Network Open.*

eTable 3. Percentage of patients by absolute error between estimated LDL-C and direct LDL-C for TG 400-799 mg/dL

Estimated LDL-C (mg/dL)	<5 mg/dL error	5-9 mg/dL error	10-19 mg/dL error	20-29 mg/dL error	≥30 mg/dL error
Extended LDL-C: <40[1]	32.6 (25.9-40.0)	31.4 (24.9-38.8)	26.7 (20.6-33.9)	6.4 (3.6-11.2)	2.9 (1.2-6.8)
Extended LDL-C: <40[2]	33.0 (26.4-40.3)	31.2 (24.8-38.5)	26.1 (20.1-33.2)	6.8 (3.9-11.7)	2.8 (1.2-6.7)
Extended LDL-C: 40-69[1]	34.2 (32.6-35.9)	26.0 (24.5-27.6)	30.1 (28.5-31.7)	7.9 (7.0-8.9)	1.8 (1.4-2.3)
Extended LDL-C: 40-69[2]	33.3 (31.6-34.9)	26.8 (25.3-28.4)	30.2 (28.7-31.9)	7.7 (6.8-8.7)	2.0 (1.6-2.6)
Extended LDL-C: 70-99[1]	30.4 (29.4-31.3)	25.6 (24.7-26.5)	30.3 (29.4-31.3)	10.5 (9.9-11.1)	3.3 (2.9-3.6)
Extended LDL-C: 70-99[2]	30.3 (29.4-31.2)	25.3 (24.5-26.2)	30.0 (29.1-31.0)	10.8 (10.2-11.4)	3.6 (3.2-4.0)
Extended LDL-C: 100-129[1]	27.4 (26.6-28.2)	24.1 (23.4-24.9)	30.4 (29.6-31.3)	12.0 (11.4-12.6)	6.0 (5.6-6.5)
Extended LDL-C: 100-129[2]	27.0 (26.2-27.8)	24.3 (23.6-25.1)	30.5 (29.7-31.3)	12.0 (11.4-12.6)	6.2 (5.7-6.6)
Extended LDL-C: 130-159[1]	24.8 (23.8-25.7)	21.9 (21.0-22.8)	31.1 (30.1-32.1)	13.6 (12.8-14.4)	8.7 (8.1-9.3)
Extended LDL-C: 130-159[2]	24.8 (23.9-25.8)	21.9 (21.0-22.8)	31.0 (30.0-32.0)	13.7 (13.0-14.5)	8.6 (8.0-9.3)
Extended LDL-C: 160-189[1]	22.6 (21.1-24.1)	20.5 (19.0-22.0)	29.8 (28.2-31.5)	13.8 (12.6-15.1)	13.3 (12.1-14.6)
Extended LDL-C: 160-189[2]	22.6 (21.1-24.1)	20.5 (19.0-22.0)	29.8 (28.2-31.5)	13.8 (12.6-15.1)	13.3 (12.1-14.6)
Extended LDL-C: ≥190[1]	18.4 (16.8-20.1)	17.2 (15.7-18.9)	25.6 (23.8-27.4)	14.1 (12.7-15.7)	24.7 (22.9-26.5)
Extended LDL-C: ≥190[2]	18.4 (16.8-20.1)	17.2 (15.7-18.9)	25.6 (23.8-27.4)	14.1 (12.7-15.7)	24.7 (22.9-26.5)

[1] 14 non-HDL-C categories
[2] 26 non-HDL-C categories

© 2021 Sajja A et al. *JAMA Network Open.*

eTable 4. Median difference between estimated and direct VLDL-C by TG and Non-HDL-C Strata

A. **TG strata, median (IQR) mg/dL**

**Triglyceride, mg/dL**	**Friedewald**	**Sampson**	**Martin/Hopkins**	**Extended Martin/Hopkins**
400-499	28.0 (16.4-37.6)	14.7 (5.6-22.9)	-5.1 (-14.6-3.0)	0.1 (-9.3-8.2)
500-599	37.6 (23.8-49.6)	19.0 (8.1-29.1)	-2.2 (-13.5-7.9)	-0.0 (-11.1-10.2)
600-699	48.2 (31.4-62.2)	23.0 (10.4-34.7)	1.2 (-11.8-13.3)	-0.1 (-13.2-11.8)
700-799	60.1 (41.8-76.0)	26.8 (14.2-39.7)	6.7 (-6.6-19.5)	0.4 (-12.6-13.6)
Overall	33.0 (20.0-46.0)	17.1 (6.9-26.7)	-3.3 (-13.7-6.2)	0.1 (-10.4-9.3)

© 2021 Sajja A et al. *JAMA Network Open.*

B. Non-HDL-C strata, median (IQR) mg/dL

Non-HDL-C, mg/dL	Friedewald	Sampson	Martin/Hopkins	Extended Martin/Hopkins
<100	57.0 (45.8-73.4)	20.5 (13.4-29.5)	0.4 (-7.3-9.4)	-0.1 (-7.0-7.7)
100-129	46.6 (36.4-59.6)	17.2 (9.4-25.2)	-1.6 (-9.3-6.9)	0.5 (-7.1-8.3)
130-159	40.0 (30.4-52.0)	16.4 (7.8-24.7)	-1.6 (-10.4-6.6)	0.5 (-8.2-8.5)
160-189	34.2 (24.0-45.8)	16.3 (6.9-25.4)	-3.3 (-12.8-5.7)	-0.1 (-9.9-8.5)
190-219	29.2 (17.8-41.6)	17.6 (6.3-27.4)	-3.5 (-14.7-6.3)	0.1 (-11.2-9.6)
220+	18.8 (3.8-32.2)	18.0 (4.0-30.0)	-6.5 (-21.7-5.6)	-0.4 (-15.8-11.7)
Overall	33.0 (20.0-46.0)	17.1 (6.9-26.7)	-3.3 (-13.7-6.2)	0.1 (-10.4-9.3)

© 2021 Sajja A et al. *JAMA Network Open.*

eTable 5. Relative Difference Between Estimated and Direct VLDL-C by TG and Non-HDL-C Strata

A. TG strata, median (IQR), %

Triglyceride, mg/dL	Friedewald	Sampson	Martin/Hopkins	Extended Martin/Hopkins
400-499	46.4 (23.1-73.7)	24.7 (8.1-44.1)	-8.5 (-21.0-5.9)	0.2 (-13.4-15.9)
500-599	53.3 (28.3-84.0)	27.0 (9.8-48.5)	-3.2 (-16.5-13.2)	-0.0 (-13.6-17.0)
600-699	60.3 (32.5-92.8)	28.7 (11.2-50.7)	1.5 (-12.6-19.6)	-0.2 (-14.5-17.2)
700-799	67.7 (39.0-104.1)	31.2 (13.8-53.0)	7.6 (-6.6-26.3)	0.5 (-12.9-18.3)
Overall	51.0 (26.2-80.6)	26.1 (9.2-46.4)	-5.0 (-18.3-10.8)	0.1 (-13.5-16.5)

© 2021 Sajja A et al. *JAMA Network Open.*

B. Non-HDL-C strata, median (IQR), %

Non-HDL-C, mg/dL	Friedewald	Sampson	Martin/Hopkins	Extended Martin/Hopkins
<100	140.5 (101.8-203.3)	49.9 (27.8-84.6)	1.1 (-15.2-27.5)	-0.2 (-14.8-21.9)
100-129	93.8 (67.1-130.6)	33.5 (16.4-56.9)	-3.1 (-16.5-15.3)	0.9 (-12.0-19.7)
130-159	70.9 (47.9-98.8)	28.5 (11.9-48.5)	-2.9 (-16.0-13.0)	0.8 (-12.2-16.8)
160-189	53.8 (33.4-78.3)	25.4 (9.4-45.2)	-5.1 (-17.6-10.1)	-0.2 (-13.1-15.4)
190-219	42.5 (22.4-65.2)	25.1 (7.9-44.1)	-5.0 (-18.4-10.2)	0.1 (-13.6-15.7)
220+	23.3 (4.0-44.7)	21.9 (4.2-41.8)	-8.0 (-22.4-8.0)	-0.6 (-15.8-16.7)
Overall	51.0 (26.2-80.6)	26.1 (9.2-46.4)	-5.0 (-18.3-10.8)	0.1 (-13.5-16.5)

© 2021 Sajja A et al. *JAMA Network Open.*

eFigure 1- TG:VLDL-C Ratio by Triglyceride and Non-HDL-C Strata

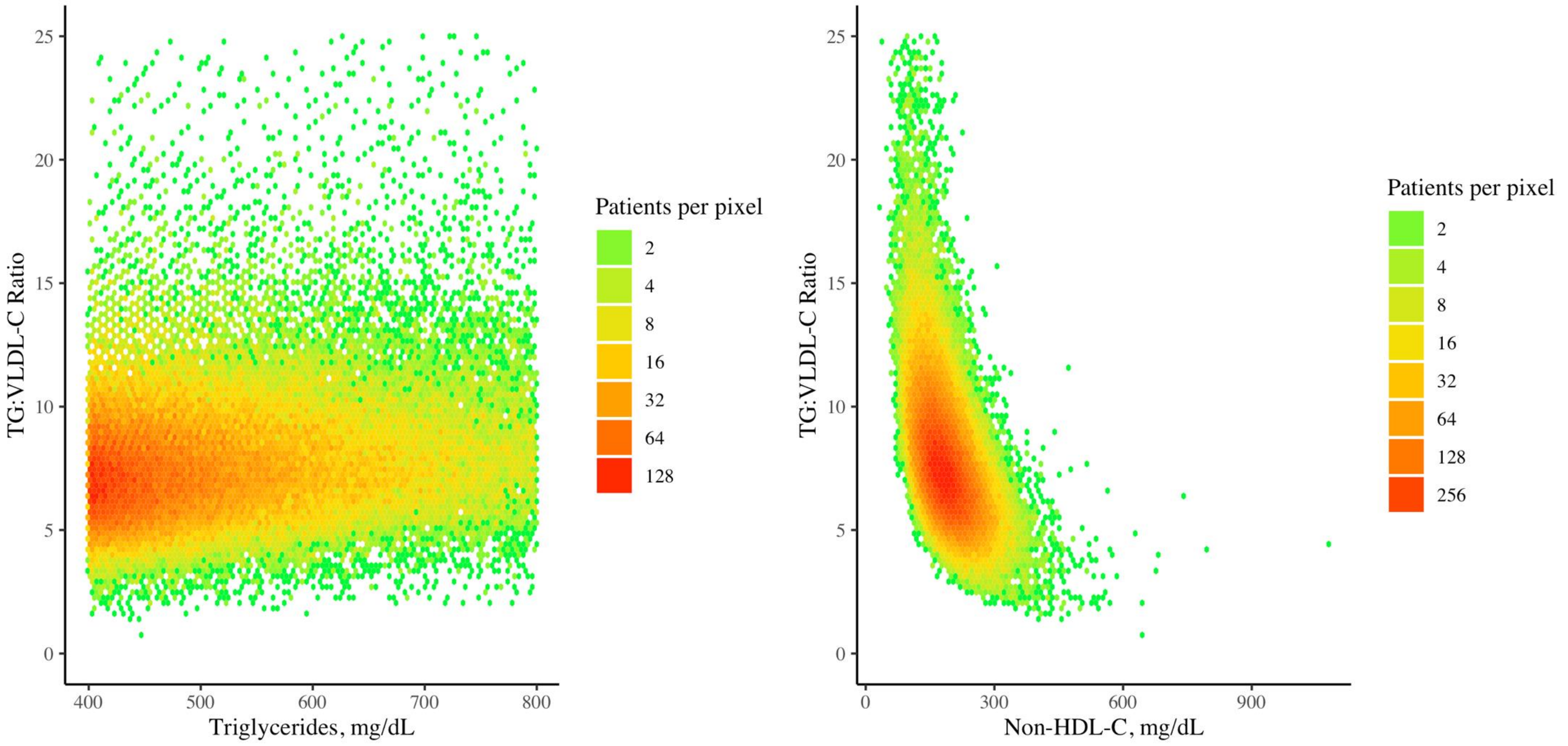

© 2021 Sajja A et al. *JAMA Network Open.*

eFigure 2- Accuracy Between Methods in Classifying LDL-C Lower than 40 mg/dL

A: Accuracy by Triglycerides Strata

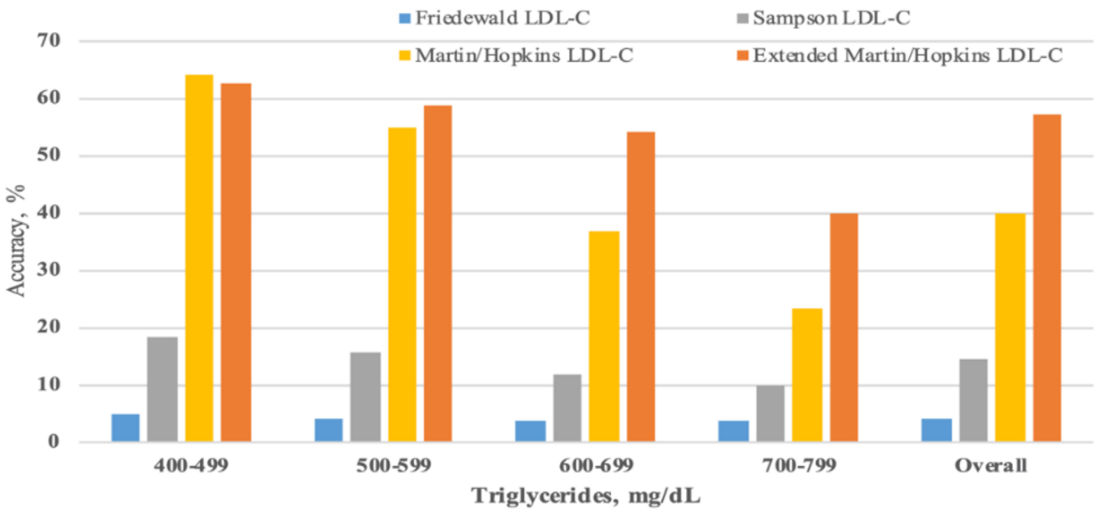

B: Accuracy by Non-HDL-C Strata

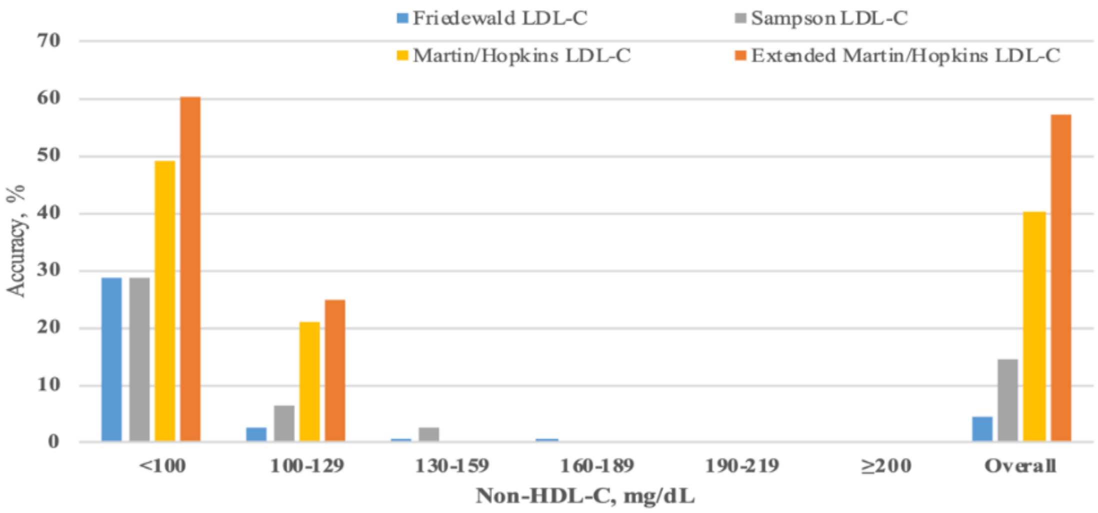

© 2021 Sajja A et al. *JAMA Network Open.*

eFigure 3- VLDL-C by Triglyceride and Non-HDL-C Strata

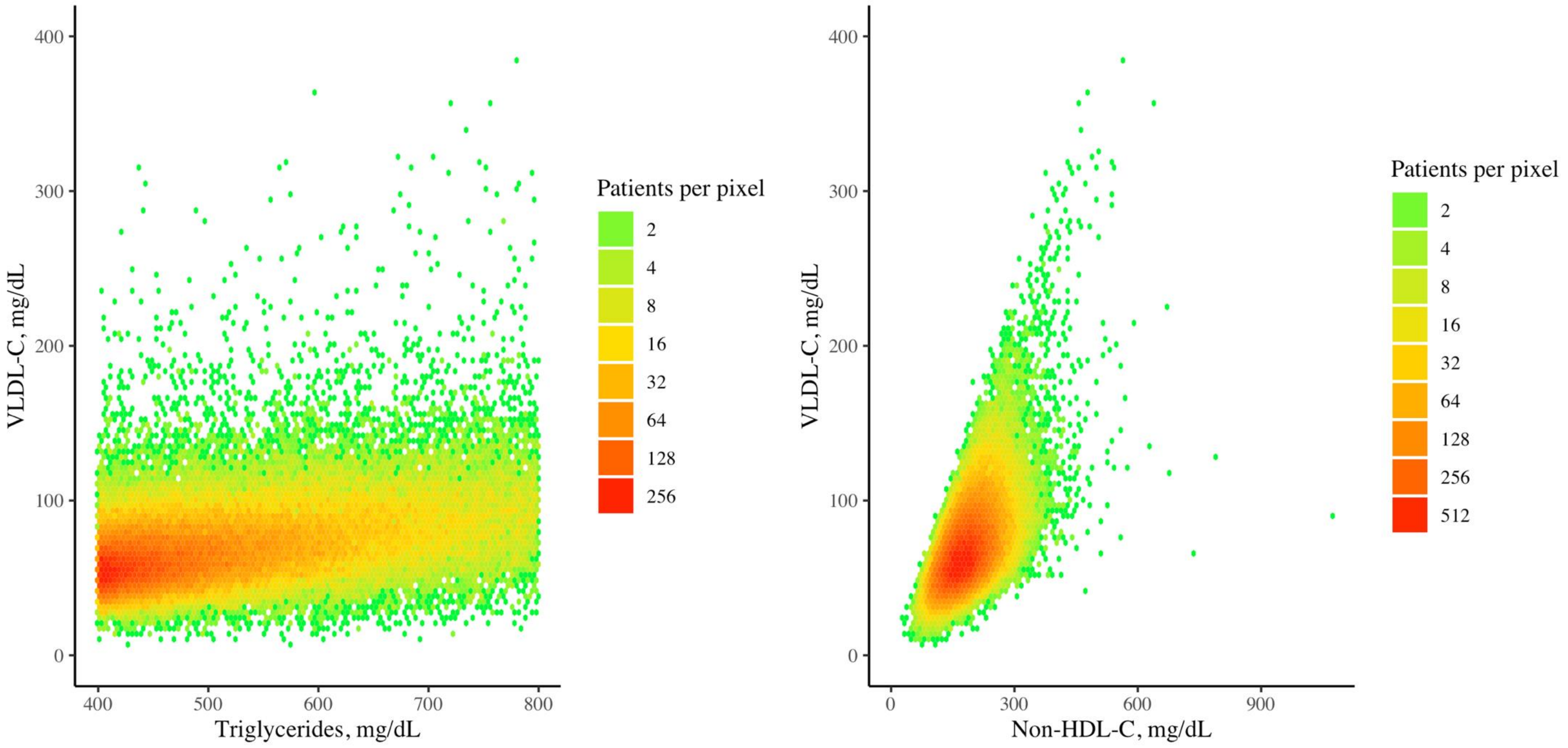

© 2021 Sajja A et al. *JAMA Network Open.*

eFigure 4- Median Difference Between Estimated and Direct LDL-C Levels by TG Level

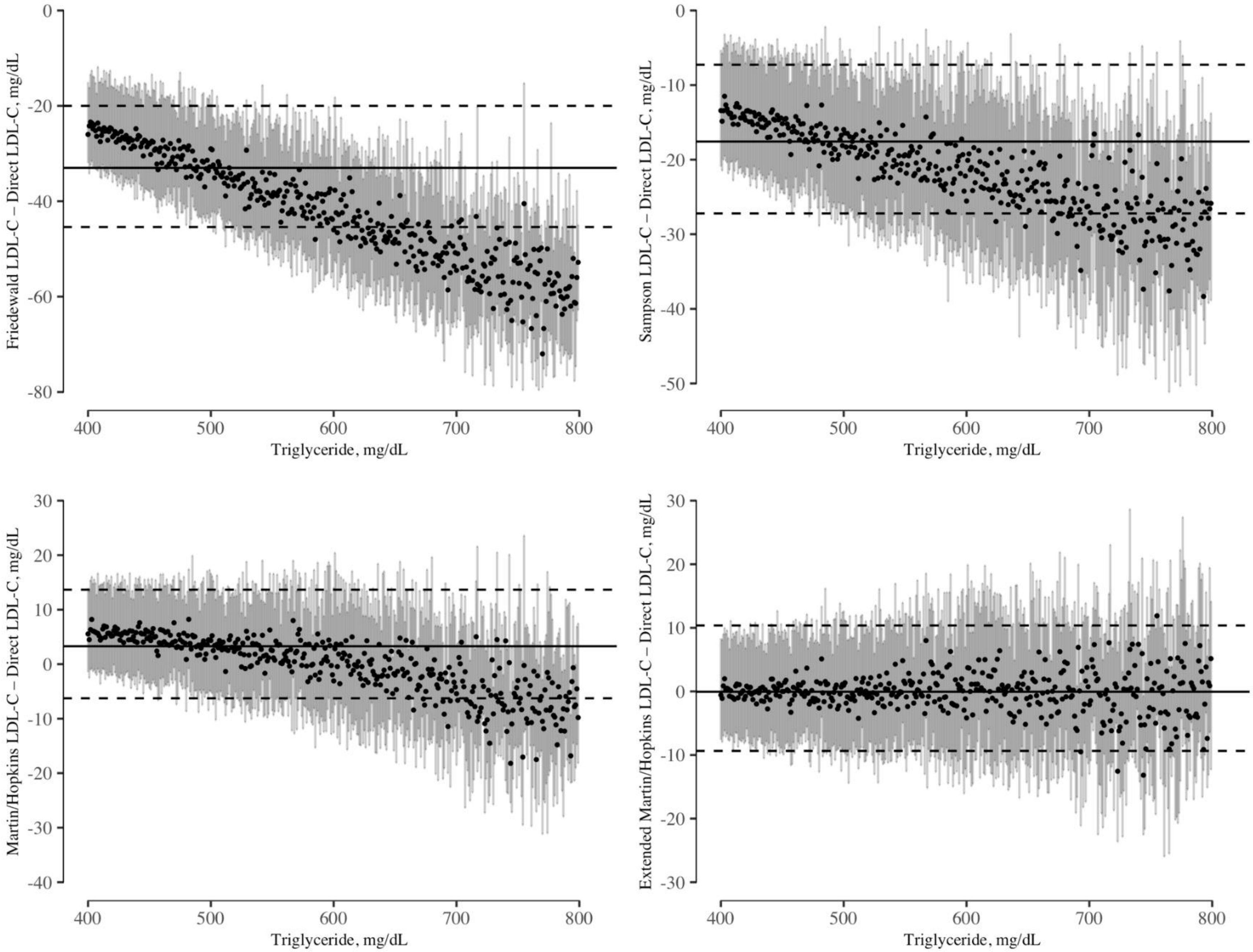

© 2021 Sajja A et al. *JAMA Network Open.*
